# Supplementary material for: Disease risk scores for skin cancers
Source: Nat Commun. 2021 Jan 8;12:160. doi: 10.1038/s41467-020-20246-5 (PMC7794415; doi:10.1038/s41467-020-20246-5)

Supplementary information for

Disease risk scores for skin cancers

Pierre Fontanillas, Babak Alipanahi, Nicholas A. Furlotte, Michaela Johnson, Catherine Wilson, 23andMe Research Team, Steve Pitts, Robert Gentleman, Adam Auton

Supplementary Table 1: Training and validation set statistics and demographics.

Percentages are computed for each column based on the sample size of the column. Longitudinal data were collected with a yearly followup survey in 2018 and 2019.

		TRAINING SET			VALIDATION SET					
					At baseline			*Prospective*		
		All	Females	Males	All	Females	Males	All	Females	Males
**Sample size**		**103,008**	57.4%	42.6%	**88,924**	61.1%	38.9%	**49,501**	63.9%	36.1%
**BCC**		14.5%	13.7%	15.5%	18.2%	16.9%	20.1%	18.8%	17.3%	21.4%
**SCC**		7.3%	6.6%	8.1%	8.9%	8.1%	10.2%	9.3%	8.4%	11.0%
**Melanoma**		3.9%	3.4%	4.5%	4.7%	4.1%	5.6%	4.6%	4.0%	5.6%
**Incident BCC**		-	-	-	-	-	-	2.3%	1.9%	3.0%
**Incident SCC**		-	-	-	-	-	-	1.7%	1.4%	2.3%
**Incident Melanoma**		-	-	-	-	-	-	0.6%	0.5%	0.8%
**Family history of skin cancer**		17.7%	19.8%	14.9%	16.4%	18.3%	13.4%	17.4%	19.2%	14.2%
**Family history of other cancers**		44.6%	44.7%	44.4%	46.8%	46.4%	47.3%	48.1%	47.4%	49.3%
**Age**	30-50 years	30.6%	29.4%	32.1%	30.3%	31.9%	27.9%	27.8%	29.9%	24.1%
	50-70	47.6%	50.6%	43.4%	49.1%	50.3%	47.1%	50.5%	51.5%	48.8%
	70-90	21.9%	20.0%	24.4%	20.6%	17.8%	25.0%	21.7%	18.6%	27.1%
**BMI**	<25	38.1%	44.8%	29.1%	33.9%	41.0%	23.0%	34.5%	40.8%	23.6%
	25-30	35.3%	28.8%	43.9%	35.6%	29.8%	44.4%	35.1%	29.9%	44.3%
	30-35	16.3%	14.8%	18.3%	18.5%	16.3%	22.0%	18.1%	16.2%	21.4%
	>35	10.3%	11.5%	8.7%	12.0%	12.9%	10.7%	12.2%	13.2%	10.6%
**Education**	High school	5.0%	5.7%	4.0%	5.7%	6.4%	4.9%	5.3%	6.2%	4.0%
	College	53.7%	56.1%	50.5%	53.9%	57.6%	48.6%	54.4%	57.3%	49.8%
	Graduate	41.3%	38.2%	45.4%	40.3%	36.1%	46.5%	40.2%	36.5%	46.2%
**Smoking**	Ever	40.9%	40.0%	42.2%	42.5%	40.6%	45.3%	41.4%	39.8%	44.2%
	Current	6.4%	6.6%	6.2%	7.9%	8.2%	7.5%	6.7%	7.1%	6.0%
**Live in metropolitan area**		90.7%	89.8%	91.9%	88.4%	88.0%	89.1%	88.6%	88.0%	89.5%

Supplementary Table 2: Skin (and blood) cancer survey.

Only the questions related to skin cancer were used in this study.

Personal History of Cancer:
1) In what year were you born?
2) What is your sex?
3) Have you ever been diagnosed with or treated for any form of blood cancer including the following?
4) Have you ever been diagnosed with or treated for any form of skin cancer or skin pre-cancer including the following?
5) Have you ever had an abnormal pap smear?
6) Have you ever had 10 or more polyps in your colon?
7) Have you ever been diagnosed with or treated for another type of cancer?
8) What other form(s) of cancer were you diagnosed with or treated for?
9) Have you ever been diagnosed with or treated for another type of cancer?
10) What other form(s) of cancer were you diagnosed with or treated for?
11) Have you ever been diagnosed with any of these hereditary cancer syndromes?
12) Which of the following blood cancers were you diagnosed with or treated for?
13) Have you ever been diagnosed with or treated for basal cell carcinoma?
14) Where on your body are/were your basal cell carcinomas located?
15) What treatments have you received for basal cell carcinoma?
16) How many basal cell carcinomas have you had in the last 2 years?
17) How old were you when you were first diagnosed with basal cell carcinoma?
18) By whom were you diagnosed with basal cell carcinoma?
19) Have you ever been diagnosed with or treated for squamous cell carcinoma?
20) Where on your body are/were your squamous cell carcinomas located?
21) What treatments have you received for squamous cell carcinoma?
22) How many squamous cell carcinomas have you had in the last 2 years?
23) How old were you when you were first diagnosed with squamous cell carcinoma?
24) By whom were you diagnosed with squamous cell carcinoma?
25) Have you ever been diagnosed with or treated for melanoma?
26) Where on your body are/were your melanomas located?
27) What types of moles did your melanoma(s) develop from?
28) What treatments have you received for melanoma?
29) How many melanomas have you had in the last 2 years?
30) How old were you when you were first diagnosed with melanoma?
31) By whom were you diagnosed with melanoma?
32) Have you ever been diagnosed with or treated for actinic keratosis?
33) Where on your body are/were your actinic keratoses located?
34) Have you ever had any of the following treatments for actinic keratosis?
35) How many actinic keratoses have you had treated in the past 2 years?
36) How old were you when you were first diagnosed with actinic keratosis?
37) By whom were you diagnosed with actinic keratosis?
38) Prior to your cancer diagnosis, how would you rate your general health?
39) Prior to your cancer diagnosis, how would you rate your stress level?

Risk Factors:
1) Which of the following best describes your skin color?
2) Which best describes the natural color of your hair?
3) How much lighter does your hair become when directly exposed to sun for 3-4 hours?
4) Which best describes the amount of red in your hair?
5) Which of the following best describes your natural eye color?
6) How many freckles total do you have on your face and body?
7) How does your skin respond when directly exposed to sun for 3-4 hours?
8) Have you ever had several large moles (1/4 inch or 6mm, flat or raised)?
9) Have you ever had a mole removed or biopsied that tested positive for abnormal cells (dysplasia)?
10) How many moles do you have on your right arm?
11) In what country and postal code do you currently live?
12) In what country and postal code did you spend the majority of your adulthood, age of 18 and older?
13) In what country and postal code did you spend the majority of your childhood, before the age of 18?
14) How often did you sunbathe before the age of 30?
15) On a typical weekday, how many hours or minutes (select one) of sun exposure do you get?
16) On a typical weekend day, how many hours or minutes (select one) of sun exposure do you get?
17) How many sunburns that caused blisters have you had?
18) How many times have you used indoor tanning equipment?
19) Have you ever been employed for a year or more in a job where you worked outdoors in the sunlight?
20) How many years were you employed in a job where you worked outdoors in the sunlight?
21) Have you smoked at least 100 cigarettes in your entire life?
22) Do you typically smoke cigarettes every day, some days, or not at all?
23) Is your familys history of cancer completely unknown to you for some reason, such as being adopted?

Family History of Cancer:
1) Your mother?
2) Your father?
3) Your sibling(s)?
4) Your child(ren)?
5) How many biological siblings do you have?
6) How many biological children do you have?
7) What type(s) of cancer was your biological mother diagnosed with or treated for?
8) What type(s) of skin cancer was your biological mother diagnosed with?
9) What type(s) of cancer was your biological father diagnosed with or treated for?
10) What type(s) of skin cancer was your biological father diagnosed with?
11) How many of your biological siblings have ever had cancer?
12) What type(s) of cancer was your biological sibling(s) diagnosed with or treated for?
13) What type(s) of skin cancer was your biological sibling(s) diagnosed with?
14) How many of your biological children have ever had cancer?
15) What type(s) of cancer was your biological child(ren) diagnosed with or treated for?
16) What type(s) of skin cancer was your biological child(ren) diagnosed with?
17) Have any of your biological parents, siblings, or children ever been diagnosed with any of these hereditary cancer syndromes?

Supplementary Table 3: Additional tested phenotypes from 23andMe database.

A total of 608 phenotypes were selected, with a maximum of 50% of missing data for the participants in the training set.

Addiction:
current tobacco user
ever tobacco user
hp alcohol 2wks
hp smoked 100 cigarettes

Autoimmune:
alopecia areata
ankylosing spondylitis
antiphospholipid syndrome
any autoimmune
autoimmune hepatitis
canker sore
canker sore pastyear
canker sores qt
celiac
celiac w biopsy
crohns
crohns or ulcerative colitis
juvenile t1d
lupus
multiple sclerosis
pediatric ibd
polymyalgia rheumatica
psoriasis
psoriatic arthritis
psoriatic arthritis unfiltered
rheumatoid arthritis
sarcoidosis
scleroderma
sjogrens syndrome
t1d
ulcerative colitis
vitiligo

Blood:
anemia
blood clots
blood thinner meds
bruise easily
deep vein thrombosis
hypocoagulation
nose bleeds
pulmonary embolism

Cancer:
any cancer
any non melanoma skin cancer
any non skin cancer
any recurrent somatic
any skin cancer
barretts esophagus
basal cell carcinoma
bladder cancer
bladder cancer dx or fh
bladder cancer fh
brain cancer
brain cancer dx or fh
brain cancer fh
breast cancer
breast cancer dx or fh
breast cancer fh
breast or ovarian cancer
cervical cancer
chemotherapy meds
colon polyps
colorectal cancer
colorectal cancer dx or fh
colorectal cancer fh
dnmt3a r882x v4
er positive breast cancer
hodgkins lymphoma
hodgkins lymphoma dx or fh
hodgkins lymphoma fh
jak2 v617f cc
kidney cancer
leukemia
leukemia dx or fh
leukemia fh
lipoma
lung cancer
lung cancer dx or fh
lung cancer fh
melanoma
melanoma dx or fh
melanoma fh
mpn or v617f
non hodgkins lymphoma
non hodgkins lymphoma dx or fh
non hodgkins lymphoma fh
ovarian cancer
ovarian cancer dx or fh
ovarian cancer fh
pancreatic cancer
pancreatic cancer dx or fh
pancreatic cancer fh
pr positive breast cancer
prostate cancer dx or fh
prostate cancer fh
radiation therapy
squamous cell carcinoma
stomach cancer
stomach cancer dx or fh
stomach cancer fh
thyroid cancer
thyroid cancer dx or fh
thyroid cancer fh

Cardiovascular:
angina
any cad
any cvd
aortic aneurysm
aortic stenosis
arrhythmia
atrial fibrillation
blood pressure meds
brain aneurysm
cad
carotid artery dis
chest pain exercise
congenital heart defect
early onset heart attack
fainted giving blood
heart attack
heart failure
high blood pressure
long qt syndrome
myocarditis
orthostatic hypotension
peripheral artery dis
raynauds
stroke
varicose veins legs

Cognitive:
clap to beat
education meta
education years
math class
play music before age 8
plays a musical instrument
self rated math ability
sing back musical note

Diet:
daily fruit servings
fruit juice frequency
hp diet quality
leafy greens frequency
pastry frequency
red meat servings
vegan
vegetable servings
vegetarian
yogurt frequency

Drug side:
caffeine trouble sleeping

Drug usage:
antiinflammatory meds
current caffeine tx
daily aspirin
finasteride
heavy caffeine drinker
oral corticosteroid ever
steroid meds
vitamin use

Endocrine:
graves
hashimotos
hyperthyroidism
hypothyroidism

Eyes:
astigmatism
cataract
cry cutting onions
db retinopathy
dry eyes treatment
farsightedness
glaucoma
HIP iritis
HIP macular degen type dry
HIP macular degen type wet
myopia v hyperopia
nearsightedness
optic neuritis
retinal detachment
retinal vein occlusion
retinitis pigmentosa
strabismus

Food preference:
chocolate
dark chocolate sneeze
fresh cantaloupe yummy
fresh cucumbers yummy
licorice
liking sweets
red wine sneeze
salty tooth
spicy food liking
sweet tooth
whisky yummy

Gastrointestinal:
appendectomy or appendicitis
chronic constipation
cirrhosis
diverticulitis
elevated liver test
gallstones
gallstones or gall bladder removal
gerd broad
hemorrhoids
hernia
HIP IBS type RomeIII IBS C
HIP IBS type RomeIII IBS D
HIP IBS type RomeIII IBS M
ibs
liver cysts
pancreatitis
ulcer

Hair:
hair curl
hair loss
hair loss
prepubertal central grey streak

Immune:
adult onset asthma
allergic asthma
any allergy
any asthma
any immune
any plant allergy
aspirin allergy clean
carried epipen
cat allergy clean
cat allergy meta
chicken allergy clean
chronic hives
current asthma
dog allergy clean
dust mites allergy clean
dust mites allergy meta
eczema
eggs allergy clean
fish allergy clean
grasses allergy clean
HIP allergies
honeybee allergy clean
immunodeficiency
juvenile asthma
juvenile asthma not current
latex allergy clean
metal allergy
milk allergy clean
mold allergy clean
mosquito bite size
mosquito bites itching
mosquito combined
mosquito severe
mycin drug allergy clean
no allergies
nonallergic asthma
peanut allergy clean
penicillin allergy clean
poison oak
pollen allergy
pollen allergy clean
pollen allergy meta
red ants allergy clean
rhinitis
seasonal allergies broad
severe asthma
shellfish allergy clean
soy allergy clean
sulfa drug allergy clean
tree nuts allergy clean
trees allergy clean
wasp allergy clean
weeds allergy clean
wheat allergy clean
yellow jackets allergy clean

Infection:
bacterial meningitis
chicken pox
childhood ear infections
chronic sinus infections
cold sores
colds last year
gingivitis
hepatitis
hepatitis a
hepatitis b
hepatitis c
hwiz hepatitis ebv
intestinal parasite
lyme disease
lyme disease
malaria meds
mononucleosis
mumps
plantar warts
plantar warts
pneumonia
pneumonia frequency
positive tb test
scarlet fever
shingles
strep throat
toenail fungus
tonsillectomy
tuberculosis
urinary tract inf
uti frequency
whooping cough

Lifestyle:
breakfast frequency
hp exercise intake
married
short of breath from stairs
tanning bed use

Longevity:
centenarian family
general health
healthy old
heart metabolic disease burden
nonagenarian
nonagenarian parent
trouble walking

Metabolic:
altitude sickness
birth weight
blood sugar meds
bmi
bmi res tx
dairy products indigestion
gout
hemochromatosis
high chol
high cholesterol
high cholesterol broad
high cholesterol dx
high cholesterol meds
high plasma glucose
hyperglycemia
lactose intolerant loose
lost 20lbs
low hdl
modtosevere altitude sickness
nafld
nash
spit frequency
t2d
t2d broad
weight
weight description
weight res tx

Morphology:
braces
cleft lip
cleft palate
extra toe
forward splits
height
height res tx
overbite
third nipple
tongue curl
wiggle ears

Musculoskeletal:
back pain frequency
bunion
carpal tunnel
dupuytrens contracture
fibromyalgia
fibromyalgia chronic fatigue
fracture
ganglion cyst foot
ganglion cyst wrist
hwiz ehlers danlos
muscular dystrophy
myasthenia gravis
osteoarthritis
osteoarthritis broad
osteoporosis
osteoporosis broad
otosclerosis
pagets
scoliosis
sugery shoulder dislocation
tmj

Neurological:
alzheimers
alzheimers fh
childhood febrile seizures
chronic fatigue
cluster headaches
db neuropathy
dyslexia
epilepsy
essential tremor
HIP chronic pain
leg jiggle
lou gehrigs
memory loss
migraine diagnosis
migraine diagnosis broad
migraine ICHD
migraine ICHD with aura
migraine ICHD without aura
numb extremities
photic sneeze
prosopagnosia
restless leg syndrome
restless leg syndrome
sma dx
trigeminal neuralgia

Parkinson:
PD all pop con no fh
pd family history

Personality:
ability handle stress
adventurous
amount of stress
appreciates art music or literature
can be cold and aloof
can be moody
can be somewhat careless
can be tense
car accidents
center of attention
coffee spilled anger
comfort taking risks
criticized anger
cry easily
cut off freeway anger
desk clean
does a thorough job
does things efficiently
extraversion combined
fear of speaking
generates a lot of enthusiasm
gets nervous easily
has a forgiving nature
has an active imagination
has an assertive personality
has few artistic interests
heated argments frequency
hothead
interruptions anger
is considerate and kind to almost everyone
is depressed often feels sad
is easily distracted
is emotionally stable not easily upset
is full of energy
is generally trusting
is ingenious a deep thinker
is inventive
is original comes up with new ideas
is outgoing sociable
is relaxed handles stress well
is reserved
is sometimes rude to others
is sometimes shy inhibited
is talkative
left out social activity
life satisfaction
life satisfaction
likes to cooperate with others
likes to reflect play with ideas
makes plans and follows through with them
nail biting frequency
narcissism
organizer
perseveres until the task is finished
personality activity
personality aesthetics
personality agreeableness
personality altruism
personality anxiety
personality assertiveness
personality compliance
personality conscientiousness
personality depression
personality extraversion
personality ideas
personality neuroticism
personality openness
personality order
personality self discipline
prefers work that is routine
recharge by socializing
remains calm in tense situations
se failure
se positive attitude
se satisfaction with self
self rated attractiveness
shyness
social support
stammer
starts quarrels with others
talk versus listen
tends to be disorganized
tends to be lazy
tends to be quiet
tends to find fault with others
values artistic aesthetic experiences
worries a lot

Pigmentation:
age gray
any freckles
eyecolor
eyecolor simple
freckle combined qt
gray amount
haircolor
haircolor rough
red hair amount
red hair cc
red hair vs blond and light brown c

Pregnancy:
gestational diabetes pc
hbp or preeclampsia pc
preeclampsia or hbp

Psychiatric:
acrophobia
adhd
adhd add meds
anxiety
autism
autism fh
autism spectrum
bipolar
depression
depression anxiety meds
eating disorder
hair pulling
mania
maternal depression
ocd
panic
phobia
ptsd
trichotillomania

Renal:
chronic kidney disease
db nephritis
HIP urinary condition
HIP urinary condition type OAB
HIP urinary condition type SUI
kidney disease
kidney infection
kidney stones

Reproductive:
endometriosis
ever pregnant
female infertility
fraternal twin
identical twin
ivf
pcos
three plus losses

Respiratory:
bronchitis frequency
chronic bronchitis
COPD
cystic fibrosis
emphysema
HIP IPF

Sensation:
ability to smell
anosmia
carsick
congenital deafness
good sense of direction
hearing aid
misophonia
motionsick combined
tinnitus

Skin:
acne as teenager
actinic keratosis
dandruff frequency
dry skin frequency
foot odor
hidradenitis
lichen planus
moles
rosacea all
severe acne
shampoo type
skin tags
stretch marks
stretch marks any
sweat during workout
sweaty feet
sweaty palms

Sleep:
avg sleep
chronotype
daytime sleepiness
insomnia
insomnia broad
insomnia narrow
morning person
narcolepsy
pee middle night
rem sleep behavior disorder dx
rem sleep disorder
sleep apnea
sleep meds
sleep needed
sleep paralysis
sleep walk cc
snore
sound sleeper
sweat while sleeping
talk in sleep

Sports:
achilles tendinitis
acl tear
elbow tendinitis
patellar tendinitis
plantar fasciitis
rotator cuff tear
shin splints

Teeth:
born with teeth
cavities many
dental superstar
flossing frequency
number of cavities
teeth grinding

Supplementary Table S4: General linear model (GLM with binomial response) results for the best model (20-risk factor model) including factors from the cancer survey.

All the linear coefficients were converted to Odds Ratios (OR) after fixing to OR=1 the first level for binary or categorical factors, or the minimum class for continuous factors. The 95% CI, P-values (: <0.05; **: <0.01; ***: <0.001), and the deviance explained are shown. For continuous factors (Age and Typical sun exposure per week), distributions were sliced per class and a OR is recomputed per class (P-value were not recomputed for each class). Models also include the five first principal components (PCs) controlling for ancestry.*

Variable	Level	BCC	SCC	Melanoma
**Age**		**9.87%**	**10.7%**	**3.27%**
	30	1	1	1
	40	3.42 [2.89-4.04]	2.52 [1.94-3.28]	1.54 [1.31-1.8]
	50	8.65 [7.12-10.5]	6.46 [4.75-8.8]	2.4 [1.99-2.9]
	60	18.01 [14.63-22.16]	16.13 [11.54-22.55]	3.78 [3.09-4.63]
	70	36.94 [29.93-45.59]	40.7 [28.9-57.33]	6.29 [5.11-7.74]
**Sex**		**0.05%**	**0.09%**	**0.21%**
	M	1	1	1
	F	0.67 *** [0.67-0.67]	0.65 *** [0.65-0.65]	0.59 *** [0.59-0.59]
**Dysplastic moles**		**3.05%**	**2.08%**	**11.91%**
	no	1	1	1
	yes	2.62 *** [2.59-2.65]	2.24 *** [2.21-2.27]	6.81 *** [6.8-6.82]
**Family history of skin cancer**		**1.33%**	**1.14%**	**0.08%**
	Risk 0	1	1	1
	Risk 1	1.54 [1.51-1.56]	1.44 [1.42-1.47]	1.18 [1.14-1.21]
	Risk 2	2.4 [2.27-2.53]	2.2 [2.08-2.33]	1.26 [1.15-1.38]
	Risk 3	3.76 [3.21-4.4]	3.27 [2.78-3.84]	1.48 [1.17-1.88]
**Skin color**		**0.8%**	**0.79%**	**0.61%**
	extremely fair	1	1	1
	light	0.94 * [0.92-0.96]	0.9 ** [0.88-0.93]	0.98 [0.94-1.01]
	medium	0.79 *** [0.78-0.8]	0.74 *** [0.73-0.75]	0.8 ** [0.79-0.81]
	olive	0.62 *** [0.59-0.64]	0.65 *** [0.61-0.68]	0.81 * [0.76-0.87]
**Freckling tendency**		**0.77%**	**0.7%**	**0.51%**
	none	1	1	1
	few	1.2 *** [1.19-1.22]	1.19 *** [1.18-1.21]	1.18 *** [1.16-1.2]
	lots	1.57 *** [1.56-1.57]	1.57 *** [1.57-1.57]	1.48 *** [1.47-1.48]
	many	2.06 *** [1.98-2.14]	1.98 *** [1.89-2.07]	1.67 *** [1.59-1.76]
**Sunbathing frequency before age of 30**		**0.9%**	**0.79%**	**0.3%**
	never	1	1	1
	rarely	1.05 [1.01-1.09]	1.03 [0.98-1.08]	0.91 [0.86-0.96]
	sometimes	1.41 *** [1.35-1.49]	1.29 *** [1.21-1.37]	1.03 [0.96-1.11]
	frequently	1.81 *** [1.73-1.89]	1.73 *** [1.63-1.84]	1.27 ** [1.18-1.35]
	very frequently	2.21 [2.14-2.28]	2.08 [1.99-2.17]	1.43 [1.37-1.5]
**Actinic keratosis before age of 40**		**0.52%**	**0.7%**	**0.03%**
	no	1	1	1
	yes	3.66 *** [3.35-4.01]	3.95 *** [3.62-4.31]	1.41 *** [1.22-1.61]
**Blisters caused by sunburns**		**0.26%**	**0.23%**	**0.07%**
	0	1	1	1
	1-2	1.13 *** [1.12-1.13]	1.22 *** [1.21-1.24]	1 [0.99-1.02]
	3-4	1.25 *** [1.25-1.26]	1.37 *** [1.36-1.39]	1.11 [1.1-1.12]
	5+	1.42 *** [1.41-1.43]	1.43 *** [1.41-1.44]	1.21 *** [1.2-1.22]
**Skin response to 3-4 hours of sun exposure**		**0.18%**	**0.17%**	**0.19%**
	always burns	1	1	1
	usually burns	0.95 [0.93-0.97]	0.93 [0.91-0.95]	0.87 [0.85-0.9]
	sometimes burns tans	0.77 [0.75-0.79]	0.74 [0.72-0.77]	0.72 [0.69-0.75]
	rarely burns	0.62 [0.62-0.63]	0.61 [0.6-0.61]	0.59 [0.59-0.59]
	never burns	0.57 [0.49-0.66]	0.59 [0.49-0.71]	0.74 [0.58-0.95]
**Typical sun exposure per week**		**0.12%**	**0.1%**	**0.07%**
	0	1	1	1
	320	1.19 [1.18-1.19]	1.2 [1.19-1.21]	1.14 [1.13-1.16]
	640	1.35 [1.34-1.36]	1.35 [1.34-1.36]	1.27 [1.26-1.29]
	960	1.49 [1.45-1.52]	1.43 [1.39-1.48]	1.39 [1.33-1.45]
	1280	1.57 [1.48-1.67]	1.44 [1.32-1.56]	1.48 [1.34-1.63]
	1600	1.6 [1.4-1.81]	1.36 [1.14-1.62]	1.54 [1.25-1.88]
	1920	1.56 [1.25-1.96]	1.21 [0.88-1.65]	1.56 [1.09-2.24]
**Hair color**		**0.08%**	**0.02%**	**0.25%**
	light blond	1	1	1
	dark blond	0.93 [0.9-0.96]	0.94 [0.9-0.98]	0.8 [0.77-0.84]
	red	1.02 [1.02-1.02]	1.05 [1.05-1.05]	1.13 [1.13-1.13]
	light brown	0.83 [0.8-0.87]	0.89 [0.85-0.94]	0.73 [0.69-0.77]
	dark brown	0.8 [0.76-0.83]	0.87 [0.83-0.92]	0.67 [0.63-0.71]
	black	0.72 *** [0.69-0.74]	0.78 * [0.75-0.82]	0.7 ** [0.66-0.75]
**Eye color**		**0.05%**	**0.08%**	**0.02%**
	blue	1	1	1
	blue gray	0.89 [0.87-0.9]	0.87 [0.85-0.89]	0.94 [0.91-0.96]
	gray	0.71 ** [0.59-0.86]	0.78 [0.61-0.99]	1 [0.75-1.33]
	gray green	0.91 [0.89-0.93]	0.86 [0.84-0.88]	1.01 [0.98-1.04]
	green	0.97 [0.96-0.98]	0.97 [0.96-0.98]	1 [0.98-1.01]
	light brown	0.88 [0.84-0.91]	0.9 [0.85-0.94]	0.9 [0.85-0.97]
	medium brown	0.87 [0.86-0.88]	0.83 [0.82-0.84]	0.92 [0.91-0.94]
	dark brown black	0.85 [0.82-0.88]	0.8 [0.76-0.84]	0.85 [0.8-0.91]
**Presence of large moles**		**0.03%**	**0.02%**	**0.2%**
	no	1	1	1
	yes	1.14 *** [1.13-1.14]	1.09 ** [1.08-1.1]	1.33 *** [1.32-1.34]
**Moles in your right arm**		**0.03%**	**0.07%**	**0.52%**
	less than 3	1	1	1
	3 to 6	0.91 [0.9-0.93]	0.89 [0.86-0.91]	1.14 [1.11-1.18]
	7 to 11	0.9 [0.87-0.94]	0.85 [0.81-0.89]	1.36 [1.3-1.43]
	12 to 18	0.84 [0.8-0.89]	0.76 [0.7-0.82]	1.51 [1.42-1.6]
	19 to 28	0.98 [0.91-1.05]	0.82 [0.74-0.91]	1.55 [1.42-1.68]
	29 to 39	0.84 [0.75-0.93]	0.75 [0.64-0.88]	1.64 [1.45-1.85]
	more than 39	0.98 [0.89-1.09]	0.79 [0.67-0.92]	2.11 [1.9-2.33]
**Sun hair lightening**		**0.02%**	**0.03%**	**0.02%**
	very much	1	1	1
	somewhat	0.96 [0.92-1]	0.91 [0.86-0.96]	0.89 [0.83-0.94]
	a little bit	0.98 [0.93-1.03]	0.95 [0.89-1.01]	0.96 [0.89-1.04]
	unchanged	1.07 [1.02-1.12]	1.04 [0.98-1.1]	1.01 [0.94-1.08]
**Skin response to sun exposure without sun protection**		**0.02%**	**0.02%**	**0.01%**
	painful blister	1	1	1
	blister	1.03 [1.03-1.04]	1 [1-1]	1.08 [1.08-1.09]
	burns sometimes	1.11 [1.08-1.15]	1.1 [1.06-1.14]	1.06 [1.02-1.11]
	rarely burns	1.03 [1.02-1.05]	1.02 [1.01-1.04]	1.05 [1.02-1.09]
	never burns	0.82 [0.66-1.01]	0.77 [0.57-1.03]	1.09 [0.76-1.56]
**Tanning bed**		**0.02%**	**0.03%**	**0.03%**
	never	1	1	1
	1-4times	1.03 [1.01-1.05]	1.03 [1.01-1.06]	1.04 [1.01-1.07]
	5-10times	1.1 * [1.06-1.14]	1.06 [1-1.11]	0.99 [0.93-1.06]
	11-20times	1.14 ** [1.09-1.2]	1.11 [1.04-1.19]	1.11 [1.02-1.2]
	21-30times	1.05 [0.98-1.13]	1.07 [0.97-1.18]	0.98 [0.87-1.11]
	30+times	1.08 [1.04-1.12]	1.24 *** [1.19-1.3]	1.23 ** [1.16-1.29]
**Outdoor job**		**0.01%**	**0.01%**	**0.01%**
	no	1	1	1
	yes	1.08 ** [1.07-1.09]	1.08 * [1.06-1.1]	1.09 * [1.07-1.12]
	**Total deviance explained:**	**19.07%**	**18.76%**	**18.73%**
	Numbers of cases - controls:	*14898/88110*	*7479/95529*	*3998/99010*

Supplementary Table 5: General linear model (GLM with binomial response) results for the best model (20-risk factor model) including factors from the cancer survey, without phenotype imputation.

See Supplementary Table 4 for a detailled description. Missing phenotype data were imputed using Hmisc library in R.

Variable	Level	BCC	SCC	Melanoma
**Age**		**9.92%**	**10.48%**	**2.9%**
	30	1	1	1
	40	4.07 [3.25-5.1]	2.79 [2-3.89]	1.6 [1.29-1.98]
	50	10.75 [8.31-13.91]	6.79 [4.63-9.96]	2.45 [1.91-3.14]
	60	22.65 [17.25-29.73]	16.34 [10.82-24.69]	3.87 [2.97-5.03]
	70	48.1 [36.61-63.21]	43.09 [28.34-65.51]	6.7 [5.14-8.73]
**Sex**		**0.06%**	**0.05%**	**0.12%**
	M	1	1	1
	F	0.63 *** [0.63-0.63]	0.65 *** [0.65-0.65]	0.59 *** [0.59-0.59]
**Dysplastic moles**		**2.8%**	**1.97%**	**13.1%**
	no	1	1	1
	yes	2.5 *** [2.46-2.53]	2.18 *** [2.15-2.22]	7.67 *** [7.67-7.68]
**Family history of skin cancer**		**1.64%**	**1.37%**	**0.11%**
	Risk 0	1	1	1
	Risk 1	1.61 [1.58-1.64]	1.5 [1.48-1.54]	1.23 [1.19-1.27]
	Risk 2	2.54 [2.4-2.69]	2.24 [2.11-2.39]	1.29 [1.17-1.43]
	Risk 3	3.92 [3.31-4.66]	3.46 [2.9-4.13]	1.48 [1.13-1.95]
**Skin color**		**0.86%**	**0.9%**	**0.7%**
	extremely fair	1	1	1
	light	0.96 [0.93-0.99]	0.91 * [0.88-0.94]	1 [0.96-1.05]
	medium	0.81 *** [0.8-0.82]	0.73 *** [0.72-0.74]	0.78 ** [0.76-0.79]
	olive	0.6 *** [0.57-0.63]	0.59 *** [0.55-0.63]	0.76 * [0.7-0.82]
**Freckling tendency**		**0.85%**	**0.89%**	**0.68%**
	none	1	1	1
	few	1.22 *** [1.21-1.24]	1.23 *** [1.21-1.25]	1.25 *** [1.22-1.28]
	lots	1.61 *** [1.61-1.61]	1.73 *** [1.72-1.73]	1.62 *** [1.61-1.64]
	many	2.17 *** [2.07-2.27]	2.15 *** [2.04-2.26]	1.84 *** [1.74-1.95]
**Sunbathing frequency before age of 30**		**0.93%**	**0.9%**	**0.4%**
	never	1	1	1
	rarely	0.98 [0.94-1.03]	0.94 [0.88-1]	0.79 * [0.74-0.85]
	sometimes	1.33 *** [1.26-1.42]	1.24 ** [1.15-1.34]	0.99 [0.9-1.08]
	frequently	1.75 *** [1.65-1.85]	1.69 *** [1.57-1.82]	1.22 [1.12-1.33]
	very frequently	2.09 [2-2.18]	2.02 [1.91-2.13]	1.33 [1.25-1.41]
**Actinic keratosis before age of 40**		**0.62%**	**0.8%**	**0.02%**
	no	1	1	1
	yes	3.94 *** [3.55-4.37]	4.03 *** [3.65-4.45]	1.28 * [1.09-1.51]
**Blisters caused by sunburns**		**0.22%**	**0.22%**	**0.08%**
	0	1	1	1
	1-2	1.08 * [1.07-1.09]	1.18 ** [1.16-1.2]	0.94 [0.93-0.96]
	3-4	1.19 *** [1.19-1.2]	1.34 *** [1.32-1.36]	1.06 [1.05-1.07]
	5+	1.33 *** [1.32-1.35]	1.41 *** [1.39-1.43]	1.16 * [1.14-1.18]
**Skin response to 3-4 hours of sun exposure**		**0.21%**	**0.17%**	**0.2%**
	always burns	1	1	1
	usually burns	0.98 [0.96-1.01]	0.89 [0.87-0.92]	0.87 [0.84-0.9]
	sometimes burns tans	0.78 [0.75-0.8]	0.72 [0.69-0.74]	0.68 [0.65-0.71]
	rarely burns	0.6 [0.6-0.61]	0.59 [0.59-0.59]	0.57 [0.57-0.57]
	never burns	0.62 [0.52-0.73]	0.62 [0.49-0.79]	0.74 [0.55-1]
**Typical sun exposure per week**		**0.16%**	**0.14%**	**0.15%**
	0	1	1	1
	320	1.21 [1.2-1.22]	1.23 [1.22-1.24]	1.22 [1.2-1.24]
	640	1.42 [1.4-1.43]	1.42 [1.41-1.44]	1.43 [1.41-1.45]
	960	1.59 [1.54-1.63]	1.55 [1.49-1.6]	1.6 [1.53-1.67]
	1280	1.71 [1.6-1.83]	1.58 [1.45-1.73]	1.72 [1.55-1.91]
	1600	1.77 [1.54-2.03]	1.52 [1.26-1.83]	1.77 [1.42-2.2]
	1920	1.76 [1.38-2.24]	1.37 [0.98-1.92]	1.74 [1.17-2.6]
**Hair color**		**0.09%**	**0.02%**	**0.27%**
	light blond	1	1	1
	dark blond	0.92 [0.89-0.96]	0.93 [0.89-0.98]	0.81 [0.76-0.85]
	red	1.06 [1.06-1.06]	1.07 [1.06-1.07]	1.21 [1.2-1.21]
	light brown	0.83 [0.79-0.88]	0.92 [0.86-0.98]	0.73 [0.68-0.79]
	dark brown	0.79 [0.75-0.84]	0.9 [0.84-0.96]	0.68 [0.63-0.73]
	black	0.71 *** [0.68-0.75]	0.81 [0.76-0.86]	0.66 * [0.6-0.73]
**Eye color**		**0.04%**	**0.08%**	**0.02%**
	blue	1	1	1
	blue gray	0.88 [0.87-0.9]	0.84 [0.81-0.86]	0.94 [0.91-0.97]
	gray	0.79 [0.62-0.99]	0.84 [0.63-1.13]	1.02 [0.7-1.48]
	gray green	0.93 [0.91-0.95]	0.86 [0.84-0.89]	1.02 [0.98-1.06]
	green	0.99 [0.98-1]	0.97 [0.96-0.98]	1.02 [1-1.03]
	light brown	0.87 [0.83-0.92]	0.93 [0.87-0.98]	0.96 [0.88-1.03]
	medium brown	0.9 [0.89-0.91]	0.82 [0.81-0.83]	0.98 [0.96-1]
	dark brown black	0.86 [0.83-0.9]	0.82 [0.78-0.87]	0.86 [0.79-0.93]
**Presence of large moles**		**0.03%**	**0.02%**	**0.2%**
	no	1	1	1
	yes	1.13 *** [1.12-1.14]	1.1 ** [1.09-1.11]	1.34 *** [1.33-1.36]
**Moles in your right arm**		**0.03%**	**0.08%**	**0.57%**
	less than 3	1	1	1
	3 to 6	0.93 [0.9-0.95]	0.89 [0.86-0.92]	1.1 [1.06-1.14]
	7 to 11	0.95 [0.91-1]	0.84 [0.79-0.9]	1.36 [1.28-1.44]
	12 to 18	0.84 [0.79-0.9]	0.72 [0.65-0.79]	1.53 [1.42-1.65]
	19 to 28	1.06 [0.97-1.15]	0.82 [0.72-0.93]	1.55 [1.4-1.72]
	29 to 39	0.9 [0.79-1.03]	0.78 [0.64-0.95]	1.6 [1.37-1.86]
	more than 39	1.05 [0.93-1.19]	0.73 [0.6-0.89]	2.31 [2.06-2.6]
**Sun hair lightening**		**0.01%**	**0.02%**	**0.03%**
	very much	1	1	1
	somewhat	0.95 [0.91-1]	0.96 [0.9-1.02]	0.89 [0.82-0.95]
	a little bit	0.96 [0.91-1.02]	1 [0.92-1.07]	0.95 [0.87-1.04]
	unchanged	1.04 [0.98-1.1]	1.08 [1-1.16]	1.04 [0.96-1.13]
**Skin response to sun exposure without sun protection**		**0.01%**	**0.03%**	**0.01%**
	painful blister	1	1	1
	blister	1.01 [1.01-1.01]	0.93 [0.93-0.93]	1.04 [1.04-1.05]
	burns sometimes	1.06 [1.03-1.1]	1.04 [1-1.08]	1.02 [0.97-1.07]
	rarely burns	0.99 [0.98-1.01]	0.96 [0.93-0.98]	1.13 [1.09-1.18]
	never burns	0.77 [0.6-1]	0.6 [0.41-0.88]	1.31 [0.87-1.98]
**Tanning bed**		**0.03%**	**0.06%**	**0.06%**
	never	1	1	1
	1-4times	1.06 [1.04-1.08]	1.03 [1-1.06]	1.07 [1.04-1.11]
	5-10times	1.13 ** [1.08-1.18]	1.13 [1.07-1.19]	0.98 [0.91-1.06]
	11-20times	1.16 ** [1.09-1.23]	1.09 [1.01-1.19]	1.16 [1.05-1.27]
	21-30times	1.14 * [1.05-1.24]	1.17 [1.05-1.3]	1.1 [0.96-1.26]
	30+times	1.16 ** [1.11-1.21]	1.32 *** [1.26-1.39]	1.32 *** [1.24-1.4]
**Outdoor job**		**0.01%**	**0.02%**	**0.04%**
	no	1	1	1
	yes	1.09 ** [1.07-1.1]	1.1 * [1.08-1.13]	1.17 ** [1.14-1.2]
	**Total deviance explained:**	**19.44%**	**19.23%**	**20.08%**
	Numbers of cases - controls:	*10043/64660*	*4871/69832*	*2534/72169*

Supplementary Table 6: General linear model (GLM with binomial response) results for the best model (20-risk factor model) including factors from the cancer survey, after age matching.

See Supplementary Table 4 for a detailled description. We used semi-parametric and non-parametric matching methods implemented in MatchIt library in R, with a ratio of one case for two controls.

Variable	Level	BCC	SCC	Melanoma
**Age**		**0.35%**	**0.01%**	**0%**
	30	1	1	1
	40	1.48 [1.46-1.51]	0.78 [0.76-0.8]	0.98 [0.96-1]
	50	1.53 [1.5-1.56]	0.84 [0.82-0.87]	1.09 [1.07-1.12]
	60	1.59 [1.56-1.62]	1.05 [1.02-1.09]	1.37 [1.34-1.41]
	70	2.05 [2.01-2.1]	1.35 [1.31-1.4]	1.87 [1.82-1.91]
**Sex**		**0.14%**	**0.32%**	**0.39%**
	M	1	1	1
	F	0.6 *** [0.6-0.6]	0.53 *** [0.53-0.53]	0.49 *** [0.49-0.49]
**Dysplastic moles**		**5.82%**	**6.04%**	**23.03%**
	no	1	1	1
	yes	3.88 *** [3.88-3.88]	3.99 *** [3.99-3.99]	12.36 *** [12.35-12.36]
**Family history of skin cancer**		**2.13%**	**2.34%**	**0.47%**
	Risk 0	1	1	1
	Risk 1	1.67 [1.67-1.67]	1.71 [1.71-1.72]	1.37 [1.37-1.38]
	Risk 2	2.96 [2.95-2.97]	2.88 [2.87-2.89]	2.17 [2.15-2.19]
	Risk 3	4.94 [4.86-5.02]	4.74 [4.66-4.83]	2.92 [2.75-3.1]
**Skin color**		**1.46%**	**1.83%**	**1.37%**
	extremely fair	1	1	1
	light	0.89 *** [0.89-0.89]	0.89 * [0.89-0.89]	0.92 [0.92-0.93]
	medium	0.71 *** [0.71-0.71]	0.69 *** [0.69-0.69]	0.71 *** [0.71-0.71]
	olive	0.59 *** [0.59-0.59]	0.63 *** [0.63-0.63]	0.75 * [0.75-0.76]
**Freckling tendency**		**1.43%**	**1.81%**	**1.51%**
	none	1	1	1
	few	1.28 *** [1.28-1.28]	1.33 *** [1.33-1.33]	1.34 *** [1.34-1.34]
	lots	1.83 *** [1.83-1.83]	1.94 *** [1.94-1.94]	1.96 *** [1.96-1.96]
	many	2.57 *** [2.57-2.58]	2.75 *** [2.75-2.76]	2.64 *** [2.62-2.66]
**Sunbathing frequency before age of 30**		**1.5%**	**1.68%**	**0.77%**
	never	1	1	1
	rarely	1.1 [1.1-1.1]	1.04 [1.03-1.04]	0.95 [0.95-0.96]
	sometimes	1.53 *** [1.52-1.53]	1.4 *** [1.39-1.4]	1.19 [1.18-1.19]
	frequently	2.14 *** [2.13-2.14]	2.07 *** [2.07-2.07]	1.47 *** [1.46-1.48]
	very frequently	2.65 [2.65-2.66]	2.72 [2.71-2.72]	1.9 [1.89-1.9]
**Actinic keratosis before age of 40**		**1.63%**	**2.11%**	**1.04%**
	no	1	1	1
	yes	>100 [0->100]	>100 [0->100]	>100 [0->100]
**Blisters caused by sunburns**		**0.46%**	**0.55%**	**0.27%**
	0	1	1	1
	1-2	1.18 *** [1.18-1.18]	1.23 *** [1.23-1.23]	1.03 [1.03-1.03]
	3-4	1.3 *** [1.3-1.3]	1.41 *** [1.41-1.42]	1.26 ** [1.26-1.26]
	5+	1.55 *** [1.55-1.55]	1.64 *** [1.64-1.64]	1.44 *** [1.44-1.44]
**Skin response to 3-4 hours of sun exposure**		**0.33%**	**0.5%**	**0.33%**
	always burns	1	1	1
	usually burns	0.92 [0.92-0.92]	0.93 [0.93-0.93]	0.91 [0.91-0.91]
	sometimes burns tans	0.73 [0.73-0.73]	0.66 [0.66-0.66]	0.7 [0.7-0.7]
	rarely burns	0.56 [0.56-0.56]	0.48 [0.48-0.48]	0.57 [0.57-0.57]
	never burns	0.49 [0.49-0.5]	0.47 [0.47-0.48]	0.71 [0.69-0.73]
**Typical sun exposure per week**		**0.18%**	**0.21%**	**0.26%**
	0	1	1	1
	320	1.22 [1.22-1.22]	1.26 [1.26-1.27]	1.33 [1.33-1.33]
	640	1.42 [1.42-1.42]	1.5 [1.5-1.5]	1.66 [1.66-1.66]
	960	1.56 [1.56-1.56]	1.67 [1.66-1.67]	1.93 [1.93-1.94]
	1280	1.63 [1.62-1.63]	1.73 [1.73-1.74]	2.11 [2.09-2.13]
	1600	1.61 [1.6-1.63]	1.69 [1.67-1.71]	2.15 [2.09-2.21]
	1920	1.52 [1.49-1.55]	1.55 [1.5-1.59]	2.06 [1.92-2.2]
**Hair color**		**0.13%**	**0.08%**	**0.36%**
	light blond	1	1	1
	dark blond	0.93 [0.93-0.94]	0.93 [0.93-0.93]	0.84 [0.84-0.84]
	red	1.04 [1.04-1.04]	1.19 [1.19-1.19]	1.24 [1.24-1.24]
	light brown	0.8 [0.8-0.81]	0.86 [0.86-0.87]	0.74 [0.73-0.74]
	dark brown	0.76 [0.76-0.76]	0.84 [0.84-0.84]	0.64 [0.64-0.64]
	black	0.73 *** [0.73-0.73]	0.73 ** [0.73-0.73]	0.65 ** [0.64-0.65]
**Eye color**		**0.06%**	**0.19%**	**0.06%**
	blue	1	1	1
	blue gray	0.89 [0.89-0.89]	0.83 [0.83-0.83]	0.95 [0.94-0.95]
	gray	0.67 ** [0.66-0.68]	0.69 [0.68-0.7]	0.82 [0.78-0.85]
	gray green	0.92 [0.91-0.92]	0.79 [0.79-0.79]	1.06 [1.06-1.06]
	green	0.98 [0.98-0.98]	0.91 [0.91-0.91]	0.99 [0.99-0.99]
	light brown	0.88 [0.88-0.88]	0.76 [0.76-0.76]	0.81 [0.8-0.81]
	medium brown	0.85 [0.85-0.86]	0.78 [0.78-0.78]	0.91 [0.91-0.91]
	dark brown black	0.83 [0.83-0.83]	0.72 [0.72-0.72]	0.79 [0.79-0.8]
**Presence of large moles**		**0.05%**	**0.05%**	**0.28%**
	no	1	1	1
	yes	1.15 *** [1.15-1.15]	1.14 *** [1.14-1.14]	1.39 *** [1.39-1.39]
**Moles in your right arm**		**0.03%**	**0.09%**	**0.44%**
	less than 3	1	1	1
	3 to 6	0.91 [0.91-0.91]	0.86 [0.86-0.86]	1.2 [1.2-1.2]
	7 to 11	0.92 [0.92-0.92]	0.84 [0.84-0.85]	1.32 [1.32-1.33]
	12 to 18	0.94 [0.94-0.94]	0.79 [0.79-0.79]	1.38 [1.37-1.39]
	19 to 28	0.97 [0.96-0.97]	0.8 [0.79-0.8]	1.52 [1.51-1.53]
	29 to 39	0.93 [0.93-0.94]	0.65 [0.64-0.65]	1.57 [1.55-1.59]
	more than 39	1.14 [1.14-1.15]	1.01 [1-1.02]	1.93 [1.91-1.95]
**Sun hair lightening**		**0.05%**	**0.06%**	**0.09%**
	very much	1	1	1
	somewhat	0.92 [0.91-0.92]	0.92 [0.92-0.92]	0.82 [0.82-0.83]
	a little bit	0.96 [0.96-0.96]	0.98 [0.98-0.98]	0.94 [0.93-0.94]
	unchanged	1.08 [1.08-1.08]	1.1 [1.1-1.11]	1.07 [1.07-1.08]
**Skin response to sun exposure without sun protection**		**0.05%**	**0.05%**	**0.03%**
	painful blister	1	1	1
	blister	1.05 [1.05-1.05]	1.06 [1.06-1.06]	1.11 [1.11-1.11]
	burns sometimes	1.15 [1.15-1.15]	1.17 [1.17-1.18]	1.1 [1.1-1.1]
	rarely burns	1.01 [1.01-1.01]	1.06 [1.06-1.06]	0.96 [0.96-0.96]
	never burns	0.73 [0.72-0.75]	0.74 [0.73-0.76]	0.76 [0.72-0.8]
**Tanning bed**		**0.02%**	**0.05%**	**0.1%**
	never	1	1	1
	1-4times	1.02 [1.02-1.03]	1.09 [1.08-1.09]	1.12 [1.12-1.12]
	5-10times	1.11 * [1.11-1.12]	1.14 [1.14-1.14]	1.07 [1.07-1.08]
	11-20times	1.15 * [1.14-1.15]	1.19 * [1.18-1.19]	1.28 * [1.27-1.29]
	21-30times	1 [1-1.01]	0.98 [0.98-0.99]	0.98 [0.97-0.99]
	30+times	1.11 * [1.11-1.11]	1.21 ** [1.2-1.21]	1.44 *** [1.44-1.45]
**Outdoor job**		**0.02%**	**0.02%**	**0.03%**
	no	1	1	1
	yes	1.1 *** [1.1-1.1]	1.08 * [1.08-1.09]	1.16 ** [1.16-1.16]
	**Total deviance explained:**	**15.93%**	**17.99%**	**30.88%**
	Numbers of cases - controls:	*14672/29288*	*7307/14513*	*3933/11739*

Supplementary Table 7: General linear model (GLM with binomial response) results for the best model (20-risk factor model) including factors from the cancer survey, and the polygenic risk score (PRS).
See Supplementary Table 4 for a detailled description.

Variable	Level	BCC	SCC	Melanoma
**Age**		**9.88%**	**10.69%**	**3.27%**
	30	1	1	1
	40	3.38 [2.86-4]	2.49 [1.91-3.24]	1.51 [1.29-1.77]
	50	8.57 [7.06-10.41]	6.36 [4.67-8.65]	2.35 [1.95-2.83]
	60	17.96 [14.6-22.1]	15.83 [11.33-22.11]	3.71 [3.03-4.54]
	70	37.15 [30.11-45.84]	39.84 [28.31-56.08]	6.18 [5.02-7.6]
**Sex**		**0.05%**	**0.09%**	**0.2%**
	M	1	1	1
	F	0.68 *** [0.68-0.68]	0.66 *** [0.66-0.66]	0.6 *** [0.6-0.6]
**Polygenic risk score (PRS)**		**3.16%**	**1.6%**	**1.99%**
	-1.71	1	1	1
	-0.57	3.89 [3.66-4.14]	2.23 [2.06-2.41]	3.03 [2.72-3.37]
	0.57	15.16 [14.26-16.11]	4.96 [4.53-5.42]	9.16 [7.9-10.62]
	1.71	59.01 [58.98-59.04]	11.04 [10.86-11.22]	27.72 [26.15-29.39]
**Dysplastic moles**		**2.94%**	**2.06%**	**11.53%**
	no	1	1	1
	yes	2.63 *** [2.6-2.66]	2.25 *** [2.22-2.27]	6.75 *** [6.74-6.76]
**Family history of skin cancer**		**1.08%**	**0.97%**	**0.06%**
	Risk 0	1	1	1
	Risk 1	1.5 [1.48-1.53]	1.42 [1.4-1.45]	1.16 [1.13-1.19]
	Risk 2	2.29 [2.17-2.42]	2.16 [2.03-2.29]	1.24 [1.14-1.36]
	Risk 3	3.58 [3.05-4.21]	3.18 [2.71-3.74]	1.45 [1.15-1.84]
**Skin color**		**0.31%**	**0.38%**	**0.31%**
	extremely fair	1	1	1
	light	0.97 [0.95-0.99]	0.93 [0.91-0.96]	0.99 [0.95-1.03]
	medium	0.86 *** [0.85-0.87]	0.8 *** [0.79-0.81]	0.84 ** [0.82-0.85]
	olive	0.72 *** [0.69-0.75]	0.74 *** [0.7-0.78]	0.91 [0.86-0.97]
**Freckling tendency**		**0.39%**	**0.38%**	**0.37%**
	none	1	1	1
	few	1.13 *** [1.12-1.14]	1.13 *** [1.12-1.15]	1.16 ** [1.14-1.18]
	lots	1.36 *** [1.35-1.36]	1.39 *** [1.39-1.39]	1.42 *** [1.41-1.42]
	many	1.66 *** [1.59-1.72]	1.65 *** [1.58-1.73]	1.58 *** [1.5-1.66]
**Sunbathing frequency before age of 30**		**1%**	**0.87%**	**0.32%**
	never	1	1	1
	rarely	1.07 [1.03-1.11]	1.05 [1-1.1]	0.92 [0.87-0.98]
	sometimes	1.46 *** [1.39-1.54]	1.32 *** [1.24-1.41]	1.05 [0.98-1.13]
	frequently	1.88 *** [1.8-1.97]	1.79 *** [1.69-1.9]	1.3 ** [1.21-1.39]
	very frequently	2.35 [2.27-2.43]	2.17 [2.08-2.27]	1.48 [1.41-1.55]
**Actinic keratosis before age of 40**		**0.45%**	**0.64%**	**0.03%**
	no	1	1	1
	yes	3.45 *** [3.15-3.78]	3.75 *** [3.43-4.1]	1.38 ** [1.2-1.59]
**Blisters caused by sunburns**		**0.19%**	**0.18%**	**0.06%**
	0	1	1	1
	1-2	1.11 *** [1.1-1.12]	1.21 *** [1.2-1.23]	1 [0.98-1.01]
	3-4	1.23 *** [1.22-1.23]	1.35 *** [1.34-1.36]	1.1 [1.09-1.1]
	5+	1.38 *** [1.37-1.38]	1.39 *** [1.38-1.41]	1.19 ** [1.18-1.21]
**Skin response to 3-4 hours of sun exposure**		**0.1%**	**0.11%**	**0.14%**
	always burns	1	1	1
	usually burns	0.98 [0.96-1]	0.95 [0.93-0.98]	0.88 [0.86-0.9]
	sometimes burns tans	0.84 [0.81-0.86]	0.79 [0.77-0.82]	0.74 [0.72-0.78]
	rarely burns	0.7 [0.69-0.71]	0.66 [0.66-0.67]	0.63 [0.63-0.63]
	never burns	0.66 [0.57-0.76]	0.65 [0.54-0.79]	0.79 [0.62-1.01]
**Typical sun exposure per week**		**0.11%**	**0.1%**	**0.07%**
	0	1	1	1
	320	1.17 [1.17-1.18]	1.19 [1.18-1.2]	1.14 [1.13-1.15]
	640	1.33 [1.32-1.34]	1.34 [1.33-1.35]	1.28 [1.26-1.29]
	960	1.46 [1.43-1.5]	1.42 [1.38-1.47]	1.4 [1.35-1.46]
	1280	1.55 [1.46-1.65]	1.43 [1.32-1.55]	1.51 [1.37-1.66]
	1600	1.59 [1.39-1.81]	1.36 [1.14-1.62]	1.59 [1.3-1.95]
	1920	1.57 [1.25-1.97]	1.21 [0.89-1.66]	1.64 [1.15-2.36]
**Hair color**		**0.1%**	**0.05%**	**0.12%**
	light blond	1	1	1
	dark blond	0.91 [0.88-0.94]	0.92 [0.89-0.96]	0.81 [0.77-0.84]
	red	0.92 [0.92-0.92]	0.95 [0.95-0.95]	0.98 [0.98-0.99]
	light brown	0.79 [0.76-0.82]	0.85 [0.8-0.89]	0.75 [0.7-0.79]
	dark brown	0.73 [0.7-0.76]	0.79 [0.75-0.84]	0.71 [0.67-0.75]
	black	0.63 *** [0.61-0.65]	0.68 *** [0.65-0.71]	0.8 [0.74-0.85]
**Eye color**		**0.02%**	**0.07%**	**0.01%**
	blue	1	1	1
	blue gray	0.92 [0.91-0.93]	0.89 [0.87-0.91]	0.94 [0.91-0.96]
	gray	0.75 * [0.62-0.91]	0.81 [0.64-1.03]	1.03 [0.77-1.36]
	gray green	0.98 [0.96-1]	0.88 [0.86-0.91]	1.02 [0.99-1.06]
	green	1.05 [1.05-1.06]	1.01 [1-1.02]	1.01 [1-1.03]
	light brown	1 [0.96-1.04]	0.93 [0.88-0.97]	0.94 [0.88-1]
	medium brown	1 [0.99-1.01]	0.87 [0.86-0.88]	0.96 [0.94-0.97]
	dark brown black	1.01 [0.97-1.04]	0.85 [0.81-0.89]	0.9 [0.84-0.96]
**Presence of large moles**		**0.05%**	**0.03%**	**0.2%**
	no	1	1	1
	yes	1.17 *** [1.16-1.18]	1.12 *** [1.11-1.13]	1.33 *** [1.32-1.34]
**Moles in your right arm**		**0.02%**	**0.05%**	**0.53%**
	less than 3	1	1	1
	3 to 6	0.93 [0.91-0.95]	0.9 [0.87-0.92]	1.14 [1.11-1.17]
	7 to 11	0.93 [0.9-0.97]	0.87 [0.83-0.92]	1.37 [1.3-1.43]
	12 to 18	0.89 [0.84-0.94]	0.79 [0.73-0.85]	1.53 [1.44-1.63]
	19 to 28	1.02 [0.95-1.09]	0.85 [0.76-0.94]	1.56 [1.44-1.7]
	29 to 39	0.88 [0.79-0.99]	0.79 [0.67-0.92]	1.64 [1.45-1.84]
	more than 39	1.04 [0.94-1.15]	0.83 [0.71-0.97]	2.12 [1.91-2.35]
**Sun hair lightening**		**0.01%**	**0.02%**	**0.02%**
	very much	1	1	1
	somewhat	0.95 [0.91-0.99]	0.9 [0.86-0.95]	0.89 [0.84-0.95]
	a little bit	0.96 [0.92-1.01]	0.94 [0.88-1]	0.97 [0.9-1.04]
	unchanged	1.03 [0.98-1.08]	1.01 [0.95-1.07]	1.01 [0.95-1.09]
**Skin response to sun exposure without sun protection**		**0.03%**	**0.02%**	**0.01%**
	painful blister	1	1	1
	blister	1.04 [1.03-1.04]	1 [0.99-1]	1.08 [1.08-1.09]
	burns sometimes	1.13 [1.1-1.16]	1.1 [1.07-1.14]	1.07 [1.02-1.11]
	rarely burns	1.05 [1.04-1.07]	1.03 [1.01-1.05]	1.07 [1.03-1.1]
	never burns	0.83 [0.67-1.03]	0.79 [0.59-1.05]	1.11 [0.77-1.59]
**Tanning bed**		**0.01%**	**0.03%**	**0.03%**
	never	1	1	1
	1-4times	1.03 [1.01-1.05]	1.03 [1.01-1.05]	1.05 [1.02-1.08]
	5-10times	1.1 * [1.06-1.14]	1.05 [1-1.11]	0.99 [0.93-1.06]
	11-20times	1.13 ** [1.08-1.19]	1.1 [1.03-1.18]	1.1 [1.01-1.19]
	21-30times	1.03 [0.96-1.11]	1.06 [0.96-1.16]	0.98 [0.87-1.1]
	30+times	1.06 [1.02-1.1]	1.22 *** [1.17-1.28]	1.21 ** [1.14-1.27]
**Outdoor job**		**0.01%**	**0.01%**	**0.01%**
	no	1	1	1
	yes	1.08 ** [1.06-1.09]	1.07 * [1.05-1.09]	1.09 * [1.07-1.11]
	**Total deviance explained:**	**20.85%**	**19.33%**	**19.72%**
	Numbers of cases - controls:	*14893/88090*	*7476/95507*	*3997/98986*

Supplementary Table 8: General linear model (GLM with binomial response) results for the best model (31-risk factor model) including factors from the cancer survey, and the additional phenotypes from 23andMe database. ***See Supplementary Table 4 for a detailled description.***

Variable	Level	BCC	SCC	Melanoma
**Age**		**9.87%**	**10.7%**	**3.27%**
	30	1	1	1
	40	3.57 [3.02-4.21]	2.59 [1.99-3.36]	1.56 [1.33-1.82]
	50	9.33 [7.69-11.31]	6.79 [5-9.22]	2.45 [2.03-2.95]
	60	20.07 [16.34-24.67]	17.31 [12.41-24.15]	3.91 [3.2-4.79]
	70	42.49 [34.48-52.35]	44.57 [31.73-62.6]	6.64 [5.4-8.17]
**Sex**		**0.05%**	**0.09%**	**0.21%**
	M	1	1	1
	F	0.77 *** [0.76-0.77]	0.71 *** [0.71-0.71]	0.65 *** [0.65-0.66]
**Dysplastic moles**		**3.05%**	**2.08%**	**11.91%**
	no	1	1	1
	yes	2.57 *** [2.54-2.6]	2.19 *** [2.16-2.22]	6.76 *** [6.74-6.77]
**Family history of skin cancer**		**1.33%**	**1.14%**	**0.22%**
	Risk 0	1	1	1
	Risk 1	1.54 [1.52-1.57]	1.45 [1.42-1.48]	1.18 [1.15-1.22]
	Risk 2	2.38 [2.26-2.51]	2.18 [2.06-2.31]	1.26 [1.15-1.38]
	Risk 3	3.69 [3.16-4.32]	3.23 [2.75-3.79]	1.48 [1.17-1.87]
**Skin color**		**0.8%**	**0.79%**	**0.56%**
	extremely fair	1	1	1
	light	0.92 ** [0.9-0.94]	0.89 ** [0.86-0.91]	0.96 [0.93-1]
	medium	0.77 *** [0.76-0.78]	0.72 *** [0.71-0.73]	0.78 *** [0.77-0.8]
	olive	0.59 *** [0.57-0.61]	0.62 *** [0.58-0.65]	0.79 * [0.74-0.84]
**Freckling tendency**		**0.77%**	**0.7%**	**0.51%**
	none	1	1	1
	few	1.19 *** [1.17-1.2]	1.17 *** [1.16-1.19]	1.17 *** [1.15-1.19]
	lots	1.54 *** [1.53-1.54]	1.55 *** [1.55-1.55]	1.46 *** [1.46-1.46]
	many	2.05 *** [1.97-2.13]	1.97 *** [1.88-2.06]	1.66 *** [1.57-1.74]
**Sunbathing frequency before age of 30**		**0.9%**	**0.79%**	**0.3%**
	never	1	1	1
	rarely	1.03 [0.99-1.07]	1.02 [0.97-1.07]	0.91 [0.86-0.96]
	sometimes	1.34 *** [1.28-1.41]	1.24 *** [1.16-1.31]	1.02 [0.94-1.09]
	frequently	1.67 *** [1.6-1.75]	1.61 *** [1.52-1.71]	1.23 * [1.15-1.32]
	very frequently	2.04 [1.97-2.1]	1.92 [1.84-2]	1.39 [1.33-1.45]
**Actinic keratosis before age of 40**		**0.52%**	**0.7%**	**0.04%**
	no	1	1	1
	yes	3.38 *** [3.09-3.7]	3.64 *** [3.34-3.97]	1.38 ** [1.21-1.59]
**Adulthood latitude**		**0.29%**	**0.33%**	**0.03%**
	25	1	1	1
	35	0.77 [0.74-0.8]	0.76 [0.73-0.8]	0.95 [0.9-1.01]
	45	0.6 [0.58-0.61]	0.59 [0.57-0.61]	0.91 [0.86-0.95]
**BMI**		**0.33%**	**0.19%**	**0.01%**
	20	1	1	1
	25	0.47 [0.42-0.53]	0.52 [0.45-0.61]	0.76 [0.62-0.92]
	30	0.35 [0.31-0.39]	0.41 [0.35-0.47]	0.67 [0.55-0.8]
	35	0.26 [0.24-0.28]	0.33 [0.3-0.36]	0.59 [0.51-0.67]
**Blisters caused by sunburns**		**0.28%**	**0.23%**	**0.13%**
	0	1	1	1
	1-2	1.13 *** [1.13-1.14]	1.23 *** [1.21-1.24]	1.01 [1-1.02]
	3-4	1.26 *** [1.26-1.27]	1.37 *** [1.36-1.39]	1.12 [1.11-1.13]
	5+	1.44 *** [1.43-1.45]	1.44 *** [1.42-1.45]	1.22 *** [1.21-1.24]
**Skin response to 3-4 hours of sun exposure**		**0.19%**	**0.18%**	**0.16%**
	always burns	1	1	1
	usually burns	0.93 [0.91-0.94]	0.91 [0.89-0.94]	0.87 [0.85-0.9]
	sometimes burns tans	0.74 [0.72-0.76]	0.72 [0.7-0.75]	0.72 [0.69-0.75]
	rarely burns	0.59 [0.59-0.59]	0.58 [0.58-0.59]	0.59 [0.59-0.59]
	never burns	0.54 [0.47-0.63]	0.57 [0.47-0.69]	0.74 [0.58-0.94]
**Physical activity**		**0.06%**	**0.02%**	**0%**
	no	1	1	1
	highly active	1.12 *** [1.11-1.13]	1.06 * [1.05-1.07]	1.01 [1-1.02]
**Typical sun exposure per week**		**0.11%**	**0.1%**	**0.07%**
	0	1	1	1
	320	1.16 [1.16-1.17]	1.18 [1.17-1.2]	1.14 [1.12-1.15]
	640	1.31 [1.31-1.32]	1.33 [1.32-1.34]	1.26 [1.25-1.28]
	960	1.44 [1.41-1.48]	1.41 [1.37-1.46]	1.37 [1.32-1.43]
	1280	1.53 [1.44-1.63]	1.42 [1.31-1.54]	1.46 [1.33-1.61]
	1600	1.58 [1.4-1.79]	1.35 [1.14-1.61]	1.52 [1.25-1.86]
	1920	1.58 [1.27-1.98]	1.22 [0.9-1.66]	1.56 [1.09-2.22]
**Alcohol consumption**		**0.04%**	**0.03%**	**0.01%**
	no or (low) consumption	1	1	1
	intermediate consumption	1.15 *** [1.15-1.16]	1.12 *** [1.12-1.13]	1.05 [1.04-1.06]
	abuser	1.11 *** [1.11-1.11]	1.11 ** [1.11-1.11]	1 [0.99-1]
**Weight**		**0.07%**	**0.03%**	**0.03%**
	100	1	1	1
	201	1.84 [1.68-2.02]	1.56 [1.38-1.76]	1.51 [1.29-1.76]
	299	2.99 [2.93-3.05]	2.22 [2.16-2.28]	2.08 [2.01-2.16]
**Hair color**		**0.07%**	**0.03%**	**0.16%**
	light blond	1	1	1
	dark blond	0.93 [0.9-0.96]	0.94 [0.9-0.97]	0.8 [0.77-0.84]
	red	1.02 [1.02-1.03]	1.05 [1.05-1.05]	1.14 [1.14-1.14]
	light brown	0.84 [0.81-0.88]	0.91 [0.86-0.95]	0.74 [0.69-0.78]
	dark brown	0.82 [0.79-0.86]	0.9 [0.85-0.95]	0.68 [0.64-0.72]
	black	0.74 *** [0.72-0.77]	0.81 * [0.77-0.84]	0.71 ** [0.66-0.76]
**Eye color**		**0.04%**	**0.07%**	**0.02%**
	blue	1	1	1
	blue gray	0.89 [0.88-0.9]	0.87 [0.85-0.89]	0.94 [0.92-0.96]
	gray	0.72 ** [0.6-0.87]	0.78 [0.62-0.99]	1.01 [0.76-1.34]
	gray green	0.91 [0.9-0.93]	0.86 [0.84-0.88]	1.01 [0.98-1.04]
	green	0.96 [0.95-0.97]	0.95 [0.94-0.96]	1 [0.98-1.01]
	light brown	0.87 [0.84-0.91]	0.89 [0.85-0.93]	0.91 [0.85-0.97]
	medium brown	0.87 [0.86-0.88]	0.83 [0.82-0.84]	0.93 [0.91-0.94]
	dark brown black	0.86 [0.83-0.89]	0.8 [0.77-0.84]	0.86 [0.81-0.92]
**Presence of large moles**		**0.04%**	**0.01%**	**0.3%**
	no	1	1	1
	yes	1.15 *** [1.14-1.16]	1.1 *** [1.09-1.11]	1.33 *** [1.32-1.34]
**Moles in your right arm**		**0.03%**	**0.07%**	**0.36%**
	less than 3	1	1	1
	3 to 6	0.92 [0.9-0.94]	0.89 [0.87-0.92]	1.15 [1.12-1.18]
	7 to 11	0.91 [0.88-0.95]	0.86 [0.82-0.91]	1.38 [1.32-1.45]
	12 to 18	0.85 [0.8-0.89]	0.77 [0.71-0.83]	1.52 [1.44-1.62]
	19 to 28	0.99 [0.92-1.06]	0.83 [0.75-0.92]	1.57 [1.45-1.71]
	29 to 39	0.85 [0.76-0.95]	0.77 [0.66-0.9]	1.67 [1.48-1.88]
	more than 39	1 [0.9-1.1]	0.8 [0.69-0.93]	2.15 [1.94-2.38]
**Childhood latitude**		**0.02%**	**0.03%**	**0.02%**
	25	1	1	1
	35	0.9 [0.86-0.93]	0.87 [0.83-0.91]	0.89 [0.83-0.95]
	45	0.81 [0.78-0.84]	0.76 [0.73-0.79]	0.79 [0.75-0.84]
**Sun hair lightening**		**0.02%**	**0.02%**	**0.02%**
	very much	1	1	1
	somewhat	0.96 [0.92-1]	0.91 [0.87-0.96]	0.88 [0.83-0.94]
	a little bit	0.97 [0.93-1.02]	0.95 [0.89-1.01]	0.96 [0.89-1.03]
	unchanged	1.06 [1.01-1.11]	1.04 [0.98-1.1]	1 [0.94-1.07]
**Skin response to sun exposure without sun protection**		**0.02%**	**0.02%**	**0.01%**
	painful blister	1	1	1
	blister	1.02 [1.01-1.02]	0.99 [0.99-0.99]	1.08 [1.07-1.08]
	burns sometimes	1.1 [1.07-1.13]	1.1 [1.06-1.14]	1.05 [1.01-1.1]
	rarely burns	1.03 [1.02-1.05]	1.04 [1.02-1.05]	1.05 [1.02-1.08]
	never burns	0.83 [0.67-1.03]	0.78 [0.59-1.05]	1.09 [0.76-1.55]
**Tanning bed**		**0.02%**	**0.04%**	**0.04%**
	never	1	1	1
	1-4times	1.04 [1.02-1.06]	1.04 [1.02-1.07]	1.05 [1.02-1.08]
	5-10times	1.12 ** [1.09-1.16]	1.08 [1.03-1.13]	1 [0.94-1.07]
	11-20times	1.17 *** [1.12-1.23]	1.15 * [1.07-1.22]	1.12 [1.03-1.21]
	21-30times	1.08 [1.01-1.16]	1.1 [1-1.21]	1 [0.89-1.12]
	30+times	1.12 ** [1.08-1.16]	1.28 *** [1.23-1.34]	1.25 *** [1.18-1.31]
**Outdoor job**		**0.01%**	**0.01%**	**0.01%**
	no	1	1	1
	yes	1.09 *** [1.08-1.11]	1.09 ** [1.07-1.11]	1.1 * [1.08-1.12]
**Smoking**		**0.02%**	**0%**	**0.01%**
	no	1	1	1
	yes	0.93 *** [0.92-0.93]	0.97 [0.97-0.98]	0.95 [0.94-0.96]
**Adulthood elevation**		**0.01%**	**0%**	**0%**
	18	1	1	1
	1004	1.06 [1.05-1.07]	1.03 [1.02-1.04]	0.98 [0.96-1]
	2029	1.12 [1.07-1.18]	1.06 [0.99-1.13]	0.96 [0.88-1.05]
**Morning person**		**0.02%**	**0.04%**	**0.03%**
	night	1	1	1
	morning	1.07 *** [1.07-1.08]	1.11 *** [1.11-1.12]	1.09 * [1.09-1.1]
**Desk clean**		**0.03%**	**0.02%**	**0.02%**
	very clen	1	1	1
	fairly clean	0.99 [0.97-1.01]	0.97 [0.94-0.99]	0.91 [0.89-0.94]
	fairly messy	0.92 [0.91-0.94]	0.91 [0.89-0.93]	0.9 [0.87-0.92]
	very messy	0.87 [0.87-0.87]	0.86 [0.86-0.86]	0.86 [0.86-0.86]
**Seasonal allergies**		**0.02%**	**0%**	**0.07%**
	no allergy	1	1	1
	has seasonal allergy	0.93 *** [0.93-0.93]	1.02 [1.02-1.02]	0.84 *** [0.84-0.84]
	**Total deviance explained:**	**19.99%**	**19.46%**	**18.96%**
	Numbers of cases - controls:	*14898/88110*	*7479/95529*	*3998/99010*

Supplementary Table 9: General linear model (GLM with binomial response) results for 32-risk factor models.
See Supplementary Table 4 for a detailled description and Figure 2.

Variable	Level	BCC	SCC	Melanoma
**Age**		**9.87%**	**10.69%**	**3.28%**
	30	1	1	1
	40	3.5 [2.97-4.13]	2.56 [1.97-3.32]	1.53 [1.31-1.78]
	50	9.14 [7.56-11.06]	6.69 [4.94-9.07]	2.4 [1.99-2.88]
	60	19.76 [16.13-24.22]	17.04 [12.25-23.71]	3.85 [3.16-4.71]
	70	42.1 [34.26-51.75]	43.75 [31.23-61.3]	6.59 [5.37-8.08]
**Sex**		**0.05%**	**0.09%**	**0.21%**
	M	1	1	1
	F	0.78 *** [0.78-0.79]	0.73 *** [0.72-0.73]	0.67 *** [0.66-0.67]
**Polygenic risk score (PRS)**		**3.05%**	**1.54%**	**1.86%**
	-1.71	1	1	1
	-0.57	0.88 [0.34-2.28]	2.49 [1.73-3.6]	5.38 [2.97-9.73]
	0.57	3.38 [1.29-8.85]	5.36 [3.65-7.89]	16.28 [8.65-30.66]
	1.71	7.68 [5.62-10.5]	9.98 [8.77-11.34]	27.77 [22.66-34.02]
**Dysplastic moles**		**2.95%**	**2.06%**	**11.51%**
	no	1	1	1
	yes	2.58 *** [2.55-2.61]	2.2 *** [2.17-2.23]	6.7 *** [6.68-6.71]
**Family history of skin cancer**		**1.07%**	**0.97%**	**0.17%**
	Risk 0	1	1	1
	Risk 1	1.51 [1.48-1.53]	1.43 [1.4-1.46]	1.17 [1.14-1.2]
	Risk 2	2.29 [2.17-2.4]	2.14 [2.02-2.26]	1.25 [1.14-1.36]
	Risk 3	3.52 [3.01-4.12]	3.13 [2.67-3.67]	1.46 [1.16-1.84]
**Skin color**		**0.29%**	**0.38%**	**0.27%**
	extremely fair	1	1	1
	light	0.95 [0.93-0.98]	0.92 * [0.89-0.94]	0.98 [0.95-1.02]
	medium	0.84 *** [0.83-0.85]	0.78 *** [0.77-0.79]	0.82 ** [0.81-0.84]
	olive	0.68 *** [0.66-0.71]	0.7 *** [0.67-0.74]	0.89 [0.83-0.95]
**Freckling tendency**		**0.37%**	**0.38%**	**0.37%**
	none	1	1	1
	few	1.11 *** [1.1-1.12]	1.12 *** [1.11-1.13]	1.15 ** [1.13-1.17]
	lots	1.32 *** [1.32-1.32]	1.37 *** [1.37-1.37]	1.4 *** [1.39-1.4]
	many	1.62 *** [1.56-1.69]	1.66 *** [1.58-1.73]	1.56 *** [1.48-1.64]
**Sunbathing frequency before age of 30**		**1%**	**0.87%**	**0.33%**
	never	1	1	1
	rarely	1.06 [1.02-1.1]	1.04 [0.99-1.09]	0.92 [0.87-0.97]
	sometimes	1.4 *** [1.33-1.47]	1.27 *** [1.2-1.35]	1.03 [0.96-1.11]
	frequently	1.76 *** [1.68-1.84]	1.67 *** [1.58-1.77]	1.26 ** [1.18-1.35]
	very frequently	2.18 [2.11-2.25]	2 [1.92-2.09]	1.43 [1.37-1.5]
**Actinic keratosis before age of 40**		**0.45%**	**0.64%**	**0.03%**
	no	1	1	1
	yes	3.15 *** [2.88-3.45]	3.45 *** [3.17-3.77]	1.35 ** [1.18-1.55]
**Adulthood latitude**		**0.31%**	**0.34%**	**0.03%**
	25	1	1	1
	35	0.76 [0.74-0.79]	0.76 [0.73-0.8]	0.95 [0.89-1.01]
	45	0.59 [0.57-0.6]	0.59 [0.57-0.6]	0.9 [0.86-0.94]
**BMI**		**0.33%**	**0.19%**	**0.01%**
	20	1	1	1
	25	0.47 [0.42-0.52]	0.52 [0.45-0.6]	0.76 [0.63-0.92]
	30	0.34 [0.3-0.38]	0.4 [0.35-0.47]	0.67 [0.55-0.8]
	35	0.25 [0.23-0.27]	0.32 [0.29-0.36]	0.59 [0.51-0.67]
**Blisters caused by sunburns**		**0.2%**	**0.18%**	**0.1%**
	0	1	1	1
	1-2	1.12 *** [1.11-1.13]	1.21 *** [1.2-1.23]	1 [0.99-1.01]
	3-4	1.23 *** [1.23-1.24]	1.35 *** [1.33-1.36]	1.1 [1.09-1.11]
	5+	1.39 *** [1.38-1.4]	1.4 *** [1.38-1.41]	1.2 ** [1.19-1.22]
**Skin response to 3-4 hours of sun exposure**		**0.11%**	**0.11%**	**0.11%**
	always burns	1	1	1
	usually burns	0.96 [0.94-0.98]	0.94 [0.91-0.96]	0.88 [0.85-0.9]
	sometimes burns tans	0.8 [0.78-0.83]	0.77 [0.74-0.79]	0.74 [0.71-0.77]
	rarely burns	0.67 [0.66-0.67]	0.64 [0.64-0.64]	0.63 [0.62-0.63]
	never burns	0.63 [0.55-0.73]	0.63 [0.52-0.76]	0.78 [0.61-0.99]
**Physical activity**		**0.05%**	**0.02%**	**0%**
	no	1	1	1
	highly active	1.11 *** [1.11-1.12]	1.06 [1.05-1.07]	1 [0.99-1.01]
**Typical sun exposure per week**		**0.1%**	**0.09%**	**0.08%**
	0	1	1	1
	320	1.15 [1.15-1.16]	1.18 [1.17-1.19]	1.14 [1.12-1.15]
	640	1.3 [1.29-1.31]	1.32 [1.31-1.34]	1.27 [1.25-1.28]
	960	1.42 [1.39-1.46]	1.41 [1.37-1.45]	1.39 [1.34-1.44]
	1280	1.52 [1.43-1.61]	1.42 [1.31-1.54]	1.49 [1.36-1.64]
	1600	1.58 [1.39-1.79]	1.36 [1.14-1.61]	1.58 [1.29-1.92]
	1920	1.6 [1.28-2]	1.23 [0.9-1.67]	1.64 [1.15-2.33]
**Alcohol consumption**		**0.04%**	**0.03%**	**0.01%**
	no or (low) consumption	1	1	1
	intermediate consumption	1.14 *** [1.13-1.14]	1.12 *** [1.11-1.12]	1.05 [1.04-1.06]
	abuser	1.1 *** [1.1-1.11]	1.11 ** [1.11-1.11]	0.99 [0.99-1]
**Weight**		**0.07%**	**0.03%**	**0.04%**
	100	1	1	1
	201	1.89 [1.72-2.07]	1.59 [1.41-1.8]	1.55 [1.32-1.81]
	299	3.11 [3.05-3.18]	2.3 [2.24-2.36]	2.18 [2.11-2.26]
**Hair color**		**0.09%**	**0.06%**	**0.08%**
	light blond	1	1	1
	dark blond	0.91 [0.88-0.94]	0.92 [0.89-0.96]	0.81 [0.78-0.85]
	red	0.92 [0.91-0.92]	0.95 [0.95-0.95]	0.99 [0.99-1]
	light brown	0.79 [0.76-0.83]	0.86 [0.82-0.9]	0.76 [0.71-0.8]
	dark brown	0.74 [0.72-0.78]	0.82 [0.78-0.87]	0.73 [0.68-0.77]
	black	0.64 *** [0.62-0.66]	0.7 *** [0.67-0.74]	0.81 [0.75-0.86]
**Eye color**		**0.02%**	**0.05%**	**0.01%**
	blue	1	1	1
	blue gray	0.92 [0.91-0.94]	0.89 [0.87-0.91]	0.95 [0.92-0.97]
	gray	0.78 * [0.64-0.94]	0.81 [0.64-1.02]	1.04 [0.79-1.38]
	gray green	0.99 [0.97-1]	0.88 [0.86-0.9]	1.03 [1-1.06]
	green	1.05 [1.04-1.06]	0.99 [0.98-1]	1.02 [1.01-1.03]
	light brown	1.01 [0.97-1.04]	0.92 [0.88-0.96]	0.94 [0.88-1]
	medium brown	1.02 [1.02-1.03]	0.86 [0.85-0.87]	0.96 [0.94-0.97]
	dark brown black	1.04 [1-1.07]	0.85 [0.81-0.89]	0.9 [0.85-0.96]
**Presence of large moles**		**0.06%**	**0.02%**	**0.3%**
	no	1	1	1
	yes	1.19 *** [1.18-1.2]	1.13 *** [1.12-1.14]	1.33 *** [1.32-1.35]
**Moles in your right arm**		**0.02%**	**0.05%**	**0.36%**
	less than 3	1	1	1
	3 to 6	0.93 [0.91-0.95]	0.9 [0.88-0.93]	1.14 [1.11-1.17]
	7 to 11	0.94 [0.91-0.97]	0.88 [0.84-0.93]	1.37 [1.31-1.44]
	12 to 18	0.89 [0.85-0.94]	0.79 [0.74-0.86]	1.55 [1.46-1.64]
	19 to 28	1.02 [0.95-1.1]	0.86 [0.78-0.95]	1.57 [1.45-1.71]
	29 to 39	0.9 [0.81-1]	0.81 [0.69-0.94]	1.66 [1.48-1.87]
	more than 39	1.06 [0.95-1.17]	0.84 [0.72-0.98]	2.16 [1.96-2.39]
**Childhood latitude**		**0.02%**	**0.03%**	**0.02%**
	25	1	1	1
	35	0.89 [0.86-0.93]	0.87 [0.83-0.91]	0.89 [0.83-0.94]
	45	0.8 [0.78-0.83]	0.76 [0.73-0.79]	0.79 [0.74-0.83]
**Sun hair lightening**		**0.01%**	**0.02%**	**0.02%**
	very much	1	1	1
	somewhat	0.95 [0.91-0.99]	0.91 [0.86-0.96]	0.89 [0.84-0.94]
	a little bit	0.96 [0.91-1]	0.94 [0.89-1]	0.96 [0.89-1.03]
	unchanged	1.02 [0.98-1.07]	1.01 [0.96-1.07]	1 [0.94-1.07]
**Skin response to sun exposure without sun protection**		**0.02%**	**0.02%**	**0.01%**
	painful blister	1	1	1
	blister	1.01 [1.01-1.02]	0.99 [0.98-0.99]	1.08 [1.07-1.08]
	burns sometimes	1.11 [1.08-1.14]	1.1 [1.07-1.14]	1.06 [1.02-1.11]
	rarely burns	1.05 [1.04-1.06]	1.05 [1.03-1.06]	1.06 [1.03-1.1]
	never burns	0.84 [0.68-1.04]	0.81 [0.61-1.08]	1.13 [0.79-1.6]
**Tanning bed**		**0.02%**	**0.04%**	**0.03%**
	never	1	1	1
	1-4times	1.05 [1.03-1.06]	1.04 [1.02-1.06]	1.05 [1.02-1.08]
	5-10times	1.12 ** [1.08-1.16]	1.08 [1.03-1.13]	1.01 [0.95-1.07]
	11-20times	1.16 ** [1.1-1.21]	1.13 [1.06-1.21]	1.11 [1.02-1.2]
	21-30times	1.07 [1-1.14]	1.09 [0.99-1.2]	1 [0.89-1.12]
	30+times	1.1 * [1.06-1.14]	1.26 *** [1.21-1.31]	1.23 ** [1.17-1.29]
**Outdoor job**		**0.01%**	**0.01%**	**0.01%**
	no	1	1	1
	yes	1.09 *** [1.08-1.1]	1.08 * [1.06-1.1]	1.09 * [1.07-1.11]
**Smoking**		**0.02%**	**0%**	**0.01%**
	no	1	1	1
	yes	0.92 *** [0.92-0.93]	0.97 [0.96-0.97]	0.94 [0.93-0.95]
**Adulthood elevation**		**0.01%**	**0%**	**0%**
	18	1	1	1
	1004	1.06 [1.05-1.07]	1.03 [1.01-1.04]	0.98 [0.97-1]
	2029	1.13 [1.07-1.18]	1.05 [0.99-1.12]	0.97 [0.89-1.06]
**Morning person**		**0.03%**	**0.03%**	**0.01%**
	night	1	1	1
	morning	1.1 *** [1.1-1.1]	1.1 *** [1.1-1.1]	1.04 [1.04-1.05]
**Desk clean**		**0.02%**	**0.02%**	**0.04%**
	very clen	1	1	1
	fairly clean	1.01 [1-1.03]	0.97 [0.95-0.99]	0.86 [0.84-0.89]
	fairly messy	0.96 [0.94-0.97]	0.92 [0.9-0.94]	0.85 [0.83-0.87]
	very messy	0.92 [0.92-0.92]	0.88 [0.88-0.88]	0.81 [0.81-0.81]
**Seasonal allergies**		**0.02%**	**0%**	**0.07%**
	no allergy	1	1	1
	has seasonal allergy	0.92 *** [0.92-0.93]	1.02 [1.02-1.02]	0.84 *** [0.84-0.84]
	**Total deviance explained:**	**21.65%**	**19.95%**	**19.81%**
	Numbers of cases - controls:	*14874/88026*	*7472/95428*	*3994/98906*

Supplementary Table 10: Variants identified by GWAS performed on the training set, and used in the PRS for each three skin cancer.

Variant genomic positions from hg19 build.

Basal cell carcinoma

Variant ID	Chr	Pos	Non-risk allele	Risk allele	Genes	Coefficient
rs2476601	1	114377568	A	G	PTPN22	0.0873
rs74520550	1	155006450	A	C	DCST1	-0.0754
rs60960598	1	17744532	-	T	RCC2	0.1308
rs61824911	1	228979737	A	G	RHOU,TMEM78	0.0843
rs12568757	1	150729793	A	G	CTSS	-0.0587
rs2349073	2	202186986	A	C	ALS2CR12	0.1199
rs6707137	2	88554351	A	G	THNSL2,FOXI3	0.1529
rs16982253	2	16498408	C	T	MYCN,FAM49A	0.1244
rs831544	2	74125261	A	C	ACTG2	0.0321
rs10932028	2	204793063	A	C	CTLA4,ICOS	-0.0519
rs143325404	2	38308857	C	G	CYP1B1,ATL2	0.0584
rs11720317	3	20619752	C	T	SGO1,ZNF385D	0.0381
rs13076312	3	188089254	C	T	LPP	-0.0759
rs57929572	3	71563561	C	T	FOXP1	0.1015
rs401681	5	1322087	C	T	CLPTM1L	-0.1018
rs727685	5	115968619	C	G	SEMA6A	-0.0402
rs16891982	5	33951693	C	G	SLC45A2	0.5672
rs115293868	6	32649308	A	G	HLA-DQB1,HLA-DQA2	-0.101
rs12203592	6	396321	C	T	IRF4	0.4187
rs56353819	6	91012867	C	T	BACH2,MAP3K7	-0.0794
rs2016246	7	129431529	C	T	NRF1,UBE2H	0.0254
rs73183650	7	101421000	A	T	MYL10,CUX1	-0.0988
rs62445146	7	17088083	C	G	AGR3,AHR	0.0922
rs17431544	8	77467488	G	T	HNF4G,ZFHX4	0.1676
rs3901080	8	81410116	G	T	ZBTB10	0.0764
rs28792460	8	101007669	A	G	RGS22	-0.1629
rs55987622	8	126481470	C	T	TRIB1	0.0874
rs13300853	9	16901228	C	T	BNC2,CNTLN	-0.1069
rs2518723	9	21995882	C	T	RP11-145E5.5	0.0944
rs57288860	9	106973791	-	T	SMC2,OR13F1	-0.0619
rs7067933	10	90615570	C	G	ANKRD22,STAMBPL1	0.0452
rs77097907	10	8946470	A	C	GATA3	0.1933
rs2193	11	3870799	A	G	RHOG,STIM1	0.0761
rs12575636	11	95311260	G	T	SESN3,FAM76B	0.0762
rs1126809	11	89017961	A	G	TYR	-0.171
rs11023815	11	16007239	C	G	SOX6	0.0596
rs200633324	12	52898782	-	TTC	KRT6A,KRT5	0.0951
rs75273011	12	47030440	A	T	SLC38A2,SLC38A4	-0.057
rs1577900	13	100766817	A	G	PCCA	-0.0616
rs2526887	14	71355048	A	G	MAP3K9,PCNX1	0.0307
rs1129038	15	28356859	C	T	HERC2	0.1253
rs1805007	16	89986117	C	T	MC1R	0.3374
rs6059655	20	32665748	A	G	ASIP	-0.2945
rs75653149	20	34726973	A	C	CNBD2,EPB41L1	-0.0145
rs214803	20	2290333	A	C	TGM3	0.157
rs2849699	21	43089086	C	G	TMPRSS2,RIPK4	-0.0761
rs5932909	23	130553877	A	T	IGSF1,OR13H1	0.0594

Squamous cell carcinoma

Variant ID	Chr	Pos	Non-risk allele	Risk allele	Genes	Coefficient
rs2352551	2	204794757	C	T	CTLA4,ICOS	-0.1
rs3773031	3	29535317	A	G	RP11-9J18.1,RBMS3	-0.037
rs16891982	5	33951693	C	G	SLC45A2	0.5177
rs116687190	6	32652865	C	T	HLA-DQB1,HLA-DQA2	-0.0862
rs12203592	6	396321	C	T	IRF4	0.4163
rs16939289	8	77437038	A	T	HNF4G,ZFHX4	-0.136
rs868764	9	129281648	A	G	MVB12B,LMX1B	0.0572
rs7025549	9	16881256	G	T	BNC2,CNTLN	-0.1028
rs1126809	11	89017961	A	G	TYR	-0.1575
rs896513	12	130240775	C	T	TMEM132D	-0.0072
rs201156703	16	90052186	-	TA	MC1R	-0.4271
rs12456267	18	49148119	A	T	MEX3C,DCC	-0.0717
rs75653149	20	34726973	A	C	CNBD2,EPB41L1	-0.0402
rs6059655	20	32665748	A	G	ASIP	-0.2663

Melanoma

Variant ID	Chr	Pos	Non-risk allele	Risk allele	Genes	Coefficient
rs2791368	1	245918151	C	T	SMYD3	0.0914
rs907188	1	226612826	G	T	PARP1,STUM	-0.126
rs4235327	4	25026034	G	T	LGI2	-0.0549
rs16891982	5	33951693	C	G	SLC45A2	0.4679
rs7712562	5	1296072	A	G	TERT,CLPTM1L	-0.1498
rs201414626	7	28209954	-	AT	JAZF1	-0.0635
chr9:21808605	9	21808606	-	C	MTAP,RP11-145E5.5	0.1402
rs75387125	10	89931260	C	T	PTEN,RNLS	-0.3006
rs4432037	11	57103797	C	T	SSRP1,P2RX3	0.0359
rs1126809	11	89017961	A	G	TYR	-0.139
rs4899263	14	69285264	A	G	ZFP36L1,ACTN1	-0.0431
rs35083644	14	29547008	C	G	FOXG1,PRKD1	-0.0404
rs200180665	15	94068177	-	T	AC112693.2,MCTP2	0.1404
rs4778138	15	28335820	A	G	HERC2	-0.1732
rs1805007	16	89986117	C	T	MC1R	0.4361
rs56238684	20	33236696	C	G	ASIP	-0.2889
rs75653149	20	34726973	A	C	CNBD2,EPB41L1	-0.0263
rs2824133	21	18292297	A	G	CXADR	-0.0849

Supplementary Table 11: Pairwise correlations between risk scores.

Sperman correlation coefficients are reported.

BCC	**DRS**								
BCC	0.66	**DRSA**							
BCC	0.45	0.65	**PRS**						
BCC	0.74	0.03	0.04	**Demographic**					
BCC	0.32	0.56	0.32	-0.08	**Susceptibility**				
BCC	0.2	0.31	-0.04	-0.04	-0.07	**Exposure**			
BCC	0.3	0.39	0.03	0.05	0.12	0.06	**Mole**		
BCC	0.23	0.3	0.08	0.01	0.13	0.04	0.07	**Family**	
BCC	0.12	0.2	0	0.02	-0.1	0.01	-0.01	-0.03	**Misc.**
SCC	0.96	0.55	0.32	0.8	0.31	0.17	0.26	0.2	0.07
SCC	0.6	0.91	0.5	0.04	0.62	0.3	0.35	0.3	0.14
SCC	0.36	0.51	0.7	0.04	0.35	-0.05	0.02	0.07	-0.01
SCC	0.74	0.04	0.05	1	-0.08	-0.04	0.05	0.01	0.03
SCC	0.31	0.56	0.35	-0.08	0.98	-0.08	0.11	0.13	-0.1
SCC	0.18	0.3	-0.04	-0.05	-0.05	0.97	0.06	0.04	-0.02
SCC	0.37	0.37	0.05	0.15	0.09	0.06	0.79	0.05	-0.01
SCC	0.23	0.3	0.08	0.01	0.13	0.04	0.07	1	-0.03
SCC	0.12	0.2	0	0.02	-0.09	0.03	0	-0.02	0.92
MELANOMA	0.82	0.62	0.25	0.55	0.34	0.13	0.5	0.15	0.12
MELANOMA	0.45	0.74	0.29	-0.02	0.48	0.16	0.52	0.16	0.18
MELANOMA	0.23	0.33	0.38	0.03	0.28	-0.04	0.06	0.05	-0.01
MELANOMA	0.72	0.05	0.04	0.98	-0.1	-0.06	0.04	0	0.09
MELANOMA	0.29	0.53	0.33	-0.09	0.94	-0.09	0.11	0.13	-0.09
MELANOMA	0.15	0.27	-0.04	-0.05	-0.08	0.91	0.06	0.03	-0.01
MELANOMA	0.13	0.3	-0.01	-0.12	0.1	0.05	0.63	0.07	0
MELANOMA	0.23	0.3	0.08	0.01	0.13	0.04	0.07	1	-0.03
MELANOMA	0.06	0.12	-0.01	0.01	-0.1	-0.03	-0.03	-0.05	0.76

SCC	**DRS**								
SCC	0.59	**DRSA**							
SCC	0.39	0.62	**PRS**						
SCC	0.8	0.05	0.04	**Demographic**					
SCC	0.32	0.64	0.37	-0.08	**Susceptibility**				
SCC	0.17	0.31	-0.05	-0.05	-0.06	**Exposure**			
SCC	0.36	0.38	0.05	0.15	0.09	0.06	**Mole**		
SCC	0.2	0.3	0.07	0.01	0.13	0.04	0.05	**Family**	
SCC	0.08	0.17	-0.01	0.03	-0.09	-0.01	0	-0.02	**Misc.**
MELANOMA	0.8	0.62	0.27	0.55	0.33	0.12	0.45	0.15	0.12
MELANOMA	0.4	0.74	0.33	-0.02	0.48	0.16	0.37	0.16	0.17
MELANOMA	0.24	0.37	0.48	0.03	0.29	-0.04	0.05	0.05	-0.01
MELANOMA	0.78	0.05	0.04	0.98	-0.1	-0.08	0.15	0	0.09
MELANOMA	0.29	0.6	0.37	-0.09	0.94	-0.07	0.09	0.13	-0.09
MELANOMA	0.13	0.27	-0.05	-0.06	-0.1	0.91	0.06	0.03	0.01
MELANOMA	0.06	0.23	-0.03	-0.12	0.09	0.05	0.28	0.07	0
MELANOMA	0.2	0.3	0.07	0.01	0.13	0.04	0.05	1	-0.02
MELANOMA	0.02	0.07	-0.01	0.02	-0.1	-0.05	-0.02	-0.05	0.64

MELANOMA	**DRS**								
MELANOMA	0.78	**DRSA**							
MELANOMA	0.4	0.5	**PRS**						
MELANOMA	0.55	0.01	0.02	**Demographic**					
MELANOMA	0.34	0.49	0.27	-0.11	**Susceptibility**				
MELANOMA	0.13	0.18	-0.04	-0.07	-0.1	**Exposure**			
MELANOMA	0.45	0.59	0.03	-0.13	0.09	0.05	**Mole**		
MELANOMA	0.15	0.16	0.05	0	0.13	0.03	0.07	**Family**	
MELANOMA	0.12	0.18	-0.01	0.08	-0.1	-0.02	-0.03	-0.05	**Misc.**

Supplementary Table 12: General linear model (GLM with binomial response) results for pairwise interaction analysis between risk scores.
Only interactions with P-value<0.1 are reported.

Basal cell carcinoma

	Estimate	Std. Error	z value	P value	Deviance explained
(Intercept)	-24.482	0.893	-27.41	2.0e-165	
Age	0.138	0.009	16.04	6.9e-58	9.52%
Sex	0.516	0.214	2.41	1.6e-02	0.04%
Ancestry	3.849	0.475	8.09	5.7e-16	0.58%
PRS	1.854	0.194	9.58	1.0e-21	3.41%
Family history of skin cancer	1.421	0.211	6.73	1.7e-11	1.4%
Mole	10.417	1.058	9.84	7.3e-23	3.42%
Susceptibility	9.077	0.871	10.43	1.9e-25	1.03%
Exposure	8.425	0.94	8.96	3.2e-19	1.6%
Age:Sex	-0.009	0.002	-5.04	4.6e-07	0.03%
Age:PRS	-0.007	0.002	-3.58	3.4e-04	0.01%
Age:Family history of skin cancer	-0.003	0.002	-2.11	3.5e-02	0%
Age:Mole	-0.045	0.008	-5.7	1.2e-08	0.03%
Age:Exposure	-0.032	0.011	-3.07	2.2e-03	0.01%
Sex:Family history of skin cancer	-0.112	0.035	-3.24	1.2e-03	0.02%
Sex:Mole	0.366	0.186	1.97	4.9e-02	0%
Sex:Susceptibility	-0.861	0.246	-3.5	4.6e-04	0.02%
PRS:Family history of skin cancer	-0.079	0.035	-2.27	2.3e-02	0.01%
PRS:Susceptibility	-1.043	0.243	-4.29	1.8e-05	0.02%
Family history of skin cancer:Susceptibility	-0.359	0.209	-1.72	8.5e-02	0%
Family history of skin cancer:Exposure	-0.599	0.193	-3.1	1.9e-03	0.01%
Mole:Susceptibility	-3.371	1.085	-3.11	1.9e-03	0.01%
Mole:Exposure	-3.377	1.077	-3.14	1.7e-03	0.01%

Squamous cell carcinoma

	Estimate	Std. Error	z value	P value	Deviance explained
(Intercept)	-22.98	1.522	-15.1	1.6e-51	
Age	0.124	0.006	19.41	6.4e-84	10.39%
Sex	0.222	0.226	0.98	3.3e-01	0.07%
Ancestry	12.822	2.876	4.46	8.3e-06	0.89%
PRS	0.3	0.21	1.43	1.5e-01	1.61%
Family history of skin cancer	0.516	0.032	15.88	9.1e-57	1.25%
Mole	8.511	0.79	10.77	4.8e-27	2.77%
Susceptibility	12.62	2.719	4.64	3.5e-06	1.08%
Exposure	4.703	0.312	15.09	1.8e-51	1.39%
Age:Sex	-0.011	0.002	-4.37	1.3e-05	0.04%
Age:Mole	-0.067	0.012	-5.79	6.9e-09	0.07%
Sex:Family history of skin cancer	-0.209	0.041	-5.07	3.9e-07	0.05%
Sex:Mole	0.326	0.262	1.24	2.1e-01	0%
Ancestry:Susceptibility	-16.969	5.367	-3.16	1.6e-03	0.02%
PRS:Exposure	0.66	0.402	1.64	1.0e-01	0.01%

Melanoma

	Estimate	Std. Error	z value	P value	Deviance explained
(Intercept)	-19.447	0.731	-26.62	4.4e-156	
Age	0.099	0.005	20.97	1.2e-97	3.27%
Sex	0.511	0.331	1.54	1.2e-01	0.2%
Ancestry	3.946	0.738	5.35	8.9e-08	0.2%
PRS	0.207	1.116	0.19	8.5e-01	2.03%
Family history of skin cancer	0.42	0.093	4.52	6.1e-06	0.47%
Mole	11.142	0.864	12.9	4.4e-38	11.8%
Susceptibility	4.656	0.311	14.96	1.3e-50	0.9%
Exposure	6.83	0.996	6.86	6.9e-12	0.51%
Age:Sex	-0.014	0.003	-4.98	6.3e-07	0.14%
Age:Mole	-0.07	0.007	-10.55	5.0e-26	0.38%
Sex:PRS	0.167	0.106	1.58	1.1e-01	0%
Sex:Family history of skin cancer	-0.143	0.056	-2.56	1.0e-02	0.03%
Sex:Mole	0.948	0.166	5.7	1.2e-08	0.08%
Sex:Susceptibility	-1.345	0.434	-3.1	1.9e-03	0.03%
Ancestry:PRS	3.919	2.171	1.81	7.1e-02	0.01%
PRS:Susceptibility	-2.555	0.583	-4.38	1.2e-05	0.06%
Family history of skin cancer:Mole	-0.292	0.129	-2.26	2.4e-02	0.02%
Mole:Exposure	-4.407	1.423	-3.1	2.0e-03	0.03%

Supplementary Table 13: General linear model (GLM with binomial response) results for pairwise interaction analysis using the factor with larger effect in each risk score.
Only interactions with P-value<0.1 are reported.

Basal cell carcinoma

	Estimate	Std. Error	z value	P value	Deviance explained
(Intercept)	-17.172	0.674	-25.47	3.8e-143	
Age	0.117	0.006	18.24	2.4e-74	9.52%
Sex	0.239	0.19	1.26	2.1e-01	0.04%
Ancestry	4.878	0.465	10.48	1.0e-25	0.58%
PRS	1.871	0.186	10.06	8.4e-24	3.41%
Family history of skin cancer	1.223	0.16	7.64	2.2e-14	1.4%
Dysplastic moles	2.2	0.224	9.82	9.4e-23	2.73%
Skin color	-0.6	0.083	-7.22	5.3e-13	0.28%
Sunbathing frequency before age 30	0.725	0.081	8.92	4.5e-19	1.12%
BMI	0.017	0.012	1.39	1.7e-01	0.31%
Age:Sex	-0.009	0.002	-4.69	2.7e-06	0.04%
Age:PRS	-0.007	0.002	-3.9	9.8e-05	0.01%
Age:Family history of skin cancer	-0.003	0.002	-2.19	2.8e-02	0%
Age:Dysplastic moles	-0.009	0.002	-4.71	2.5e-06	0.02%
Age:Sunbathing frequency before age 30	-0.003	0.001	-3.47	5.2e-04	0.01%
Sex:Family history of skin cancer	-0.108	0.034	-3.17	1.5e-03	0.02%
Sex:Dysplastic moles	0.179	0.047	3.78	1.6e-04	0.01%
Sex:Skin color	0.081	0.027	3	2.7e-03	0.01%
Sex:BMI	-0.012	0.004	-3.06	2.2e-03	0.02%
PRS:Family history of skin cancer	-0.091	0.033	-2.75	6.0e-03	0.01%
PRS:Dysplastic moles	-0.183	0.049	-3.76	1.7e-04	0.02%
PRS:Skin color	0.062	0.027	2.33	2.0e-02	0.01%
PRS:BMI	-0.009	0.004	-2.25	2.4e-02	0%
Family history of skin cancer:Dysplastic moles	-0.087	0.035	-2.49	1.3e-02	0.01%
Family history of skin cancer:Sunbathing frequency before age 30	-0.032	0.015	-2.13	3.3e-02	0.01%
Dysplastic moles:Skin color	0.083	0.031	2.66	7.9e-03	0%
Dysplastic moles:Sunbathing frequency before age 30	-0.08	0.022	-3.64	2.7e-04	0.02%
Sunbathing frequency before age 30:BMI	-0.004	0.002	-2.52	1.2e-02	0.01%

Squamous cell carcinoma

	Estimate	Std. Error	z value	P value	Deviance explained
(Intercept)	-10.476	0.875	-11.97	5.4e-33	
Age	0.111	0.005	21.51	1.2e-102	10.39%
Sex	0.525	0.198	2.66	7.9e-03	0.07%
Ancestry	1.522	1.465	1.04	3.0e-01	0.89%
PRS	1.164	0.105	11.1	1.3e-28	1.61%
Family history of skin cancer	0.922	0.119	7.74	9.9e-15	1.25%
Dysplastic moles	1.284	0.261	4.92	8.8e-07	1.85%
Skin color	-1.015	0.351	-2.89	3.8e-03	0.38%
Sunbathing frequency before age 30	0.266	0.013	21.2	1.0e-99	0.94%
BMI	-0.025	0.009	-2.86	4.2e-03	0.17%
Age:Sex	-0.013	0.002	-5.21	1.9e-07	0.05%
Age:Dysplastic moles	-0.014	0.003	-5.39	7.1e-08	0.05%
Age:Skin color	-0.004	0.002	-2.62	8.8e-03	0.01%
Sex:Family history of skin cancer	-0.194	0.041	-4.77	1.8e-06	0.04%
Sex:Dysplastic moles	0.135	0.059	2.3	2.1e-02	0.01%
Ancestry:Skin color	1.751	0.654	2.68	7.4e-03	0.01%
PRS:Dysplastic moles	-0.248	0.077	-3.21	1.3e-03	0.03%
Family history of skin cancer:Dysplastic moles	-0.107	0.042	-2.57	1.0e-02	0.01%
Family history of skin cancer:BMI	-0.008	0.004	-2.12	3.4e-02	0.01%
Dysplastic moles:Skin color	0.069	0.04	1.75	8.1e-02	0.01%
Dysplastic moles:BMI	0.01	0.005	1.88	6.0e-02	0.01%

Melanoma

	Estimate	Std. Error	z value	P value	Deviance explained
(Intercept)	-13.774	0.536	-25.67	2.2e-145	
Age	0.093	0.005	19.65	6.4e-86	3.27%
Sex	-0.204	0.255	-0.8	4.2e-01	0.2%
Ancestry	4.055	0.72	5.63	1.8e-08	0.2%
PRS	0.74	0.144	5.12	3.0e-07	2.03%
Family history of skin cancer	0.489	0.093	5.25	1.6e-07	0.47%
Dysplastic moles	3.933	0.208	18.9	1.1e-79	11.26%
Skin color	-0.226	0.073	-3.1	1.9e-03	0.27%
Sunbathing frequency before age 30	0.224	0.047	4.71	2.5e-06	0.31%
BMI	-0.002	0.003	-0.57	5.7e-01	0%
Age:Sex	-0.016	0.003	-5.77	8.1e-09	0.17%
Age:Dysplastic moles	-0.029	0.003	-10.31	6.6e-25	0.38%
Sex:Family history of skin cancer	-0.139	0.055	-2.52	1.2e-02	0.02%
Sex:Dysplastic moles	0.395	0.071	5.58	2.4e-08	0.09%
Sex:Skin color	0.145	0.048	3.04	2.4e-03	0.02%
PRS:Skin color	0.142	0.065	2.17	3.0e-02	0.02%
Family history of skin cancer:Dysplastic moles	-0.134	0.055	-2.43	1.5e-02	0.02%
Skin color:Sunbathing frequency before age 30	-0.03	0.021	-1.42	1.6e-01	0.01%

Supplementary Table 14: Risk factor weights for DRS and DRSA computation

Risk factor	BCC	SCC	Melanoma
Age	0.0761	0.0823	0.0461
Sex (M)	0.1526	-0.4404	0.0333
Sex (F)	-0.0909	-0.7604	-0.3717
PC1	-0.5256	0.0642	-0.9599
PC2	3.4687	4.24	2.2686
PC3	-2.2573	-2.8384	-1.2158
PC4	-2.628	-4.4661	-5.2706
PC5	0.3858	0.1474	1.2237
PRS	0.5998	0.6454	0.96
Dysplastic moles (no)	-0.1356	-0.7474	-0.4968
Dysplastic moles (yes)	0.8127	0.0403	1.4048
Family history of skin cancer (Risk 0)	-0.0756	-0.7025	-0.2294
Family history of skin cancer (Risk 1)	0.3348	-0.3458	-0.074
Family history of skin cancer (Risk 2)	0.7509	0.0578	-0.0068
Family history of skin cancer (Risk 3)	1.183	0.4386	0.1498
Skin color (extremely fair)	0.1119	-0.4854	-0.1298
Skin color (light)	0.0651	-0.5723	-0.1491
Skin color (medium)	-0.0623	-0.7397	-0.323
Skin color (olive)	-0.2665	-0.8405	-0.2489
Freckling tendency (none)	-0.1111	-0.7609	-0.3509
Freckling tendency (few)	-0.0079	-0.6488	-0.2099
Freckling tendency (lots)	0.1656	-0.4448	-0.0163
Freckling tendency (many)	0.3713	-0.257	0.0923
Sunbathing frequency before age of 30 (never)	-0.3284	-0.908	-0.2803
Sunbathing frequency before age of 30 (rarely)	-0.273	-0.8701	-0.3669
Sunbathing frequency before age of 30 (sometimes)	0.0081	-0.6658	-0.2461
Sunbathing frequency before age of 30 (frequently)	0.2344	-0.3942	-0.0475
Sunbathing frequency before age of 30 (very frequently)	0.4506	-0.2147	0.0767
Actinic keratosis before age of 40 (no)	-0.0024	-0.6406	-0.2031
Actinic keratosis before age of 40 (yes)	1.1447	0.5988	0.0996
Adulthood latitude	-0.0268	-0.0268	-0.0054
BMI	-0.0482	-0.032	-0.0256
Blisters caused by sunburns (0)	-0.1421	-0.8226	-0.2621
Blisters caused by sunburns (1-2)	-0.0302	-0.6286	-0.263
Blisters caused by sunburns (3-4)	0.0688	-0.5259	-0.1644
Blisters caused by sunburns (5+)	0.1883	-0.4889	-0.0774
Skin response to 3-4 hours of sun exposure (always burns)	0.2101	-0.3918	0.0647
Skin response to 3-4 hours of sun exposure (usually burns)	0.1688	-0.4576	-0.0673
Skin response to 3-4 hours of sun exposure (sometimes burns tans)	-0.0076	-0.6564	-0.2345
Skin response to 3-4 hours of sun exposure (rarely burns)	-0.1939	-0.8387	-0.4035
Skin response to 3-4 hours of sun exposure (never burns)	-0.2509	-0.8533	-0.1847
Physical activity (no)	-0.0563	-0.66	-0.2003
Physical activity (highly active)	0.051	-0.6043	-0.1984
Typical sun exposure per week (in min.)	1.209e-04	-1.418e-04	1.709e-04
Alcohol consumption (no or (low) consumption)	-0.0678	-0.6978	-0.2185
Alcohol consumption (intermediate consumption)	0.0619	-0.5868	-0.1698
Alcohol consumption (abuser)	0.0312	-0.5951	-0.2238
Weight	0.0051	0.0038	0.0035
Hair color (light blond)	0.2351	-0.4749	0.0574
Hair color (dark blond)	0.1371	-0.5559	-0.1532
Hair color (red)	0.1474	-0.5258	0.0489
Hair color (light brown)	0.0031	-0.6248	-0.2223
Hair color (dark brown)	-0.0594	-0.6678	-0.2616
Hair color (black)	-0.212	-0.826	-0.1578
Eye color (blue)	0.01	-0.5477	-0.178
Eye color (blue gray)	-0.069	-0.6653	-0.2334
Eye color (gray)	-0.2449	-0.7631	-0.1373
Eye color (gray green)	-0.0043	-0.6758	-0.1471
Eye color (green)	0.0577	-0.5569	-0.1599
Eye color (light brown)	0.016	-0.6324	-0.2403
Eye color (medium brown)	0.0337	-0.6953	-0.2208
Eye color (dark brown black)	0.0459	-0.7094	-0.2809
Presence of large moles (no)	-0.0426	-0.6627	-0.2921
Presence of large moles (yes)	0.1298	-0.5428	-0.0032
Moles in your right arm (less than 3)	0.0356	-0.5682	-0.3243
Moles in your right arm (3 to 6)	-0.0349	-0.6706	-0.1917
Moles in your right arm (7 to 11)	-0.0273	-0.6933	-0.0075
Moles in your right arm (12 to 18)	-0.0798	-0.7987	0.1114
Moles in your right arm (19 to 28)	0.0586	-0.7178	0.1289
Moles in your right arm (29 to 39)	-0.0735	-0.7839	0.1842
Moles in your right arm (more than 39)	0.0894	-0.743	0.4471
Childhood latitude	-0.011	-0.0138	-0.0121
Sun hair lightening (very much)	0.0345	-0.5842	-0.1591
Sun hair lightening (somewhat)	-0.0168	-0.6795	-0.2777
Sun hair lightening (a little bit)	-0.01	-0.6428	-0.1978
Sun hair lightening (unchanged)	0.0553	-0.5722	-0.155
Skin response to sun exposure without sun protection (painful blister)	-0.0592	-0.6879	-0.2536
Skin response to sun exposure without sun protection (blister)	-0.0455	-0.7017	-0.1809
Skin response to sun exposure without sun protection (burns sometimes)	0.0457	-0.5899	-0.1929
Skin response to sun exposure without sun protection (rarely burns)	-0.01	-0.6435	-0.1917
Skin response to sun exposure without sun protection (never burns)	-0.2322	-0.8985	-0.1358
Tanning bed (never)	-0.0222	-0.667	-0.2315
Tanning bed (1-4times)	0.0231	-0.6267	-0.181
Tanning bed (5-10times)	0.0933	-0.5919	-0.2249
Tanning bed (11-20times)	0.1239	-0.542	-0.1294
Tanning bed (21-30times)	0.0438	-0.5803	-0.2345
Tanning bed (30+times)	0.0713	-0.4373	-0.0243
Outdoor job (no)	-0.0058	-0.641	-0.2187
Outdoor job (yes)	0.0788	-0.5643	-0.1298
Smoking (no)	0.0468	-0.6108	-0.1744
Smoking (yes)	-0.0363	-0.6433	-0.2349
Adulthood elevation	5.775e-05	2.366e-05	-1.586e-05
Morning person (night)	-0.0362	-0.673	-0.2205
Morning person (morning)	0.0579	-0.5791	-0.1794
Desk clean (very clen)	0.035	-0.5659	-0.0581
Desk clean (fairly clean)	0.0482	-0.5996	-0.2033
Desk clean (fairly messy)	-0.0085	-0.6453	-0.2214
Desk clean (very messy)	-0.0514	-0.6953	-0.2704
Seasonal allergies (no allergy)	0.0543	-0.6346	-0.1064
Seasonal allergies (has seasonal allergy)	-0.024	-0.6147	-0.2817

Supplementary Figure 1: Maps of BCC, SCC, and melanoma distributions in USA

The maps combined all data (training and validation sets) from 23andMe skin cancer cohorts. a) map for BCC, b) map for SCC, and c) map for melanoma

a) BCC

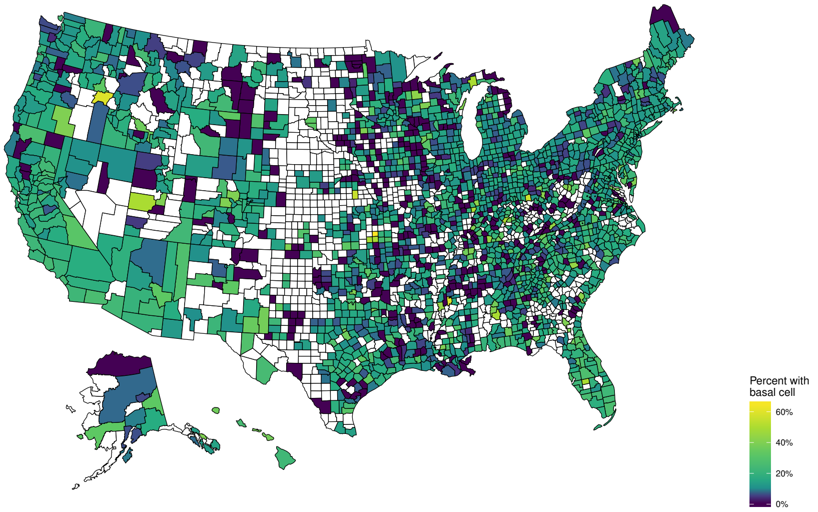

b) SCC

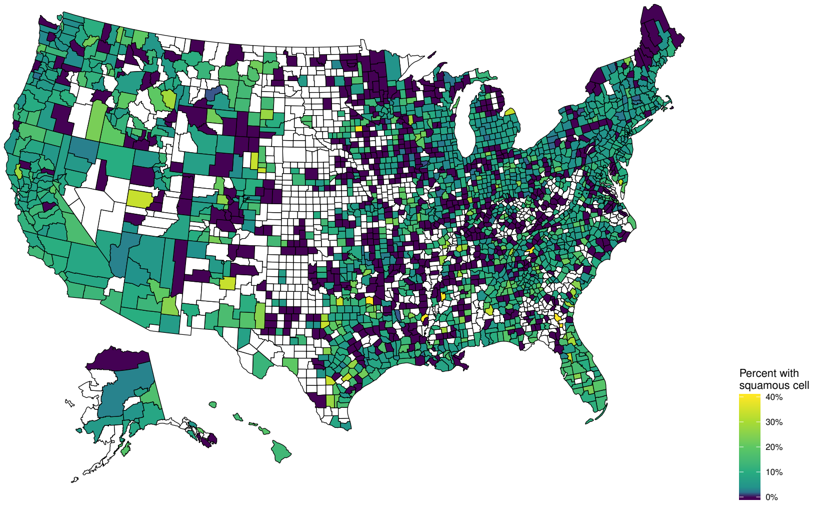

c) Melanoma

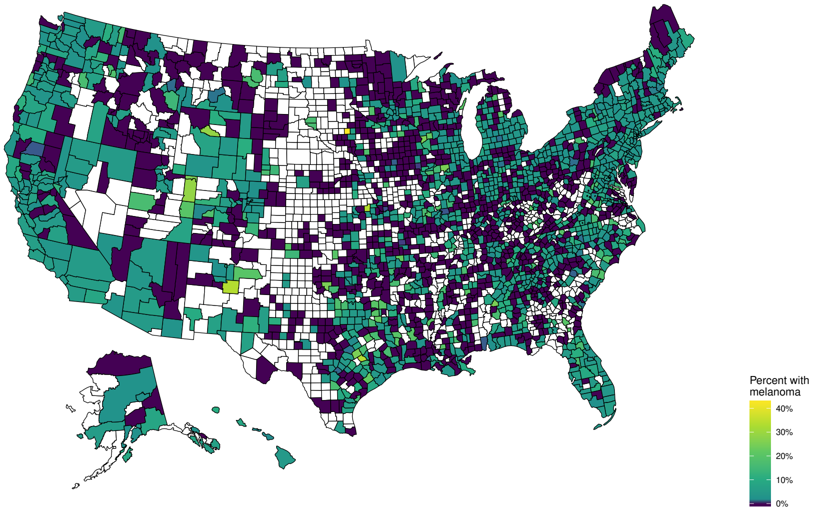

Supplementary Figure 2: Deviance explained from GLMs described in a) Supplementary Table 4 and b) Supplementary Table 8.
Stars indicate additional risk factors identified in 23andMe database.

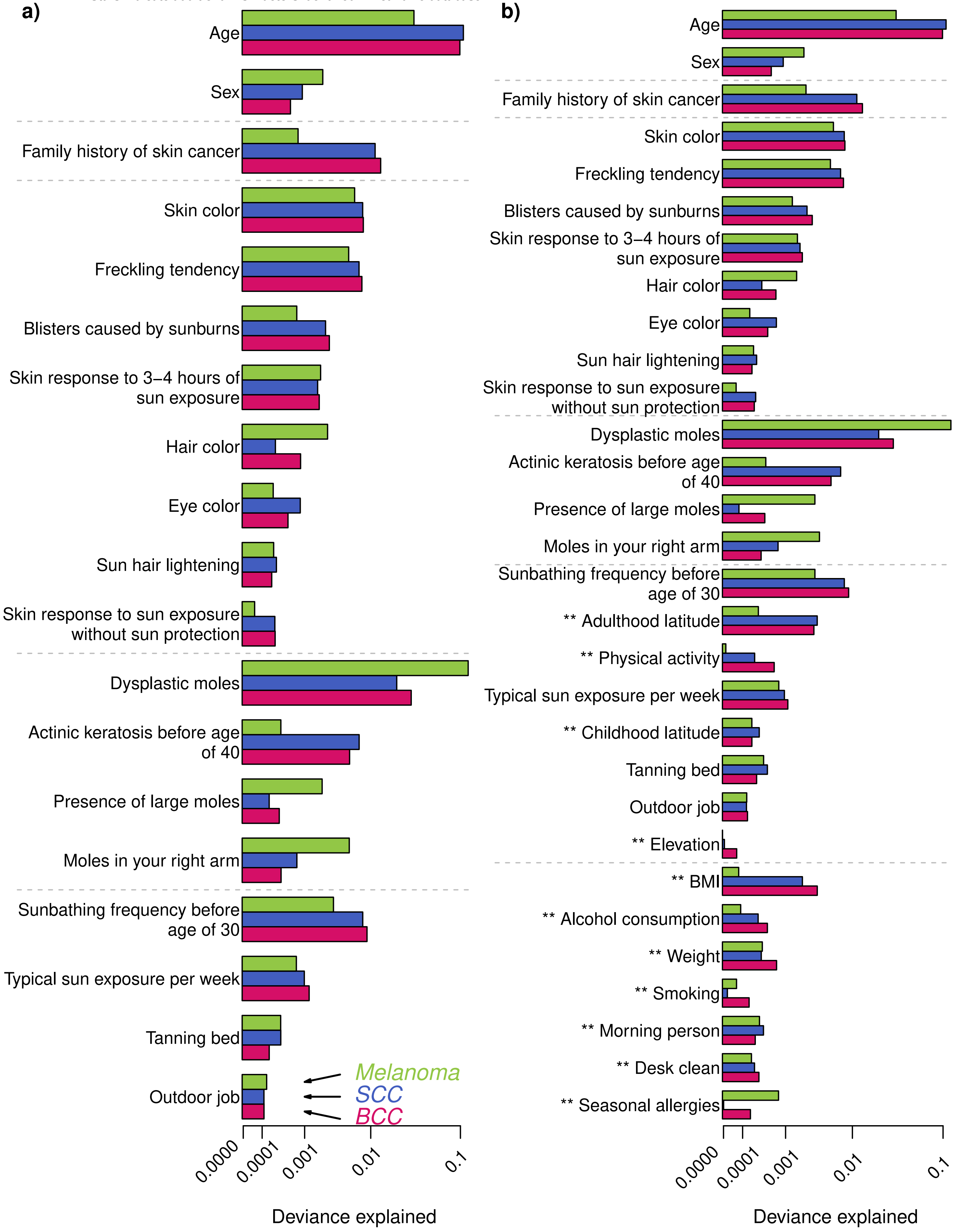

Supplementary Figure 3: Deviance explained from GLMs described in a) Supplementary Table 5 (no missing phenotype imputation) and b) Supplementary Table 6 (age matching)

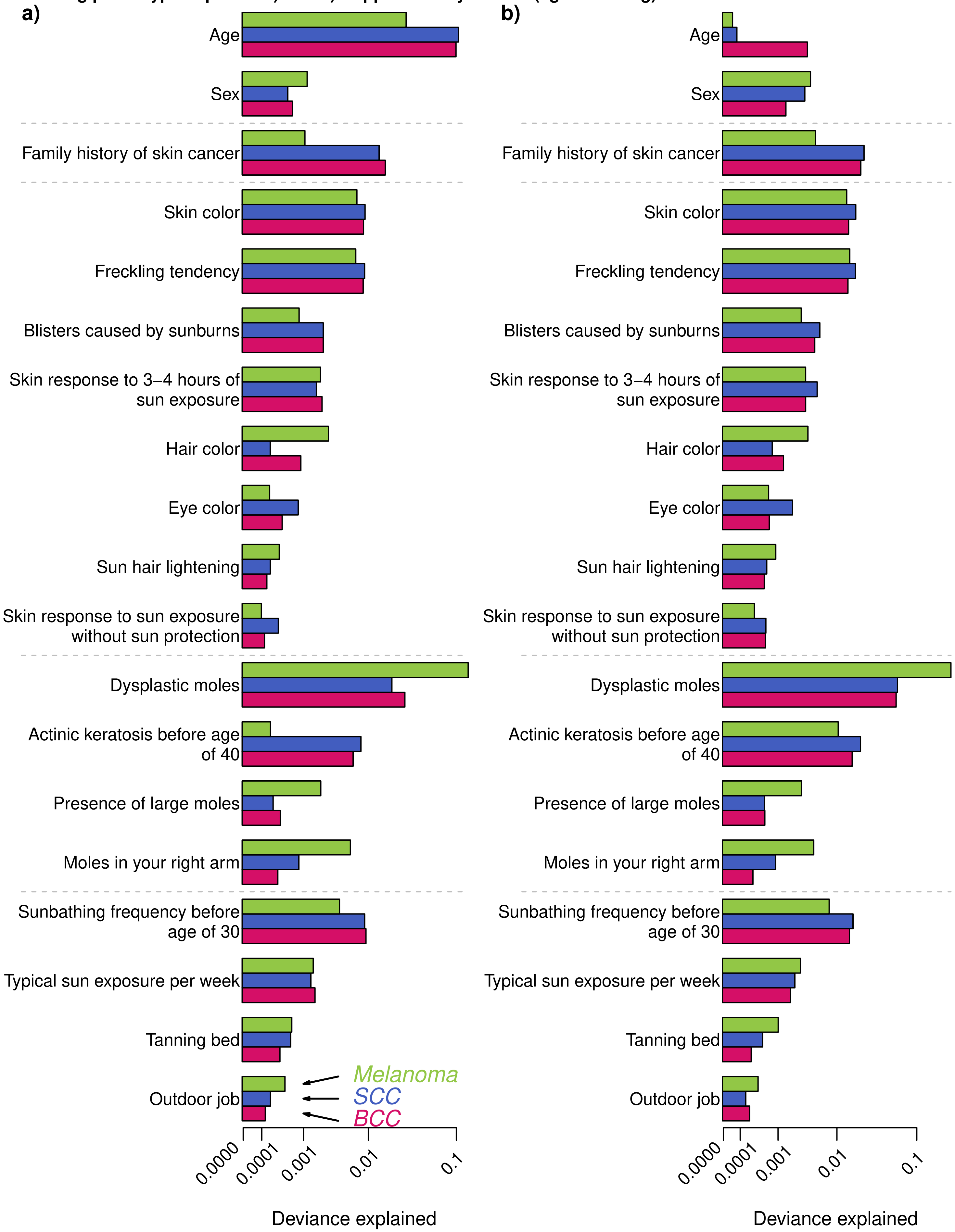

Supplementary Figure 4: Cluster analysis of additional risk factors identified in 23andMe database.

A total 608 phenotypes from 23andMe database were tested. We selected additional risk factors with P−value < 1e−8 criteria and clustered them using ClustOfVar library in R.The aggregation criterion is the decrease in homogeneity for the cluster being merged. The homogeneity of a cluster is the sum of the correlation ratio (for qualitative variables) and the squared correlation (for quantitative variables) between the variables and the center of the cluster which is the first principal component of PCAmix. PCAmix is defined for a mixture of qualitative and quantitative variables and includes ordinary principal component analysis (PCA) and multiple correspondence analysis (MCA) as special cases.

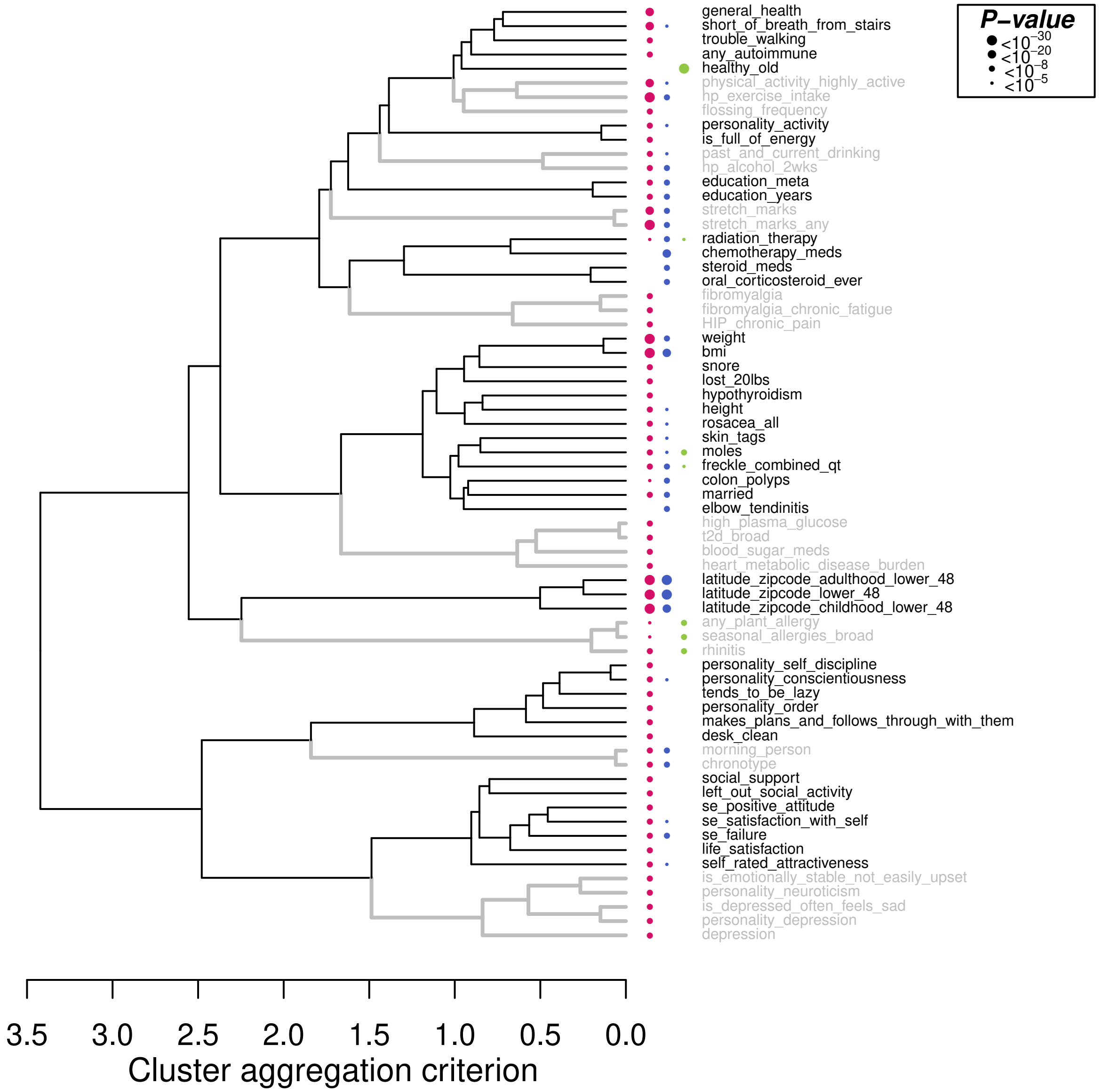

Supplementary Figure 5: Manhattan plots of each skin cancer GWAS (performed on the training set).

a) BCC GWAS, b) SCC GWAS, and c) melanoma GWAS.

a) BCC

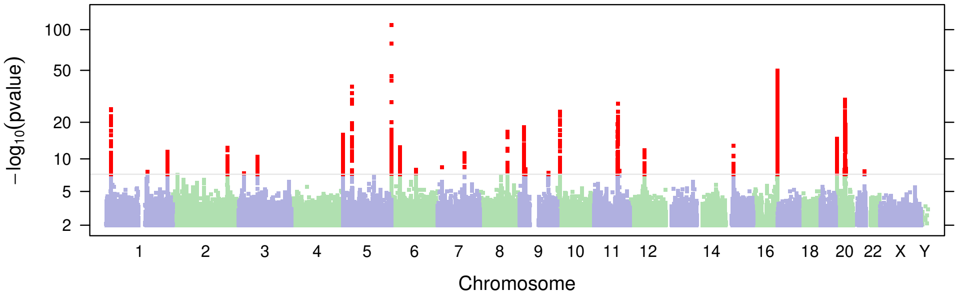

b) SCC

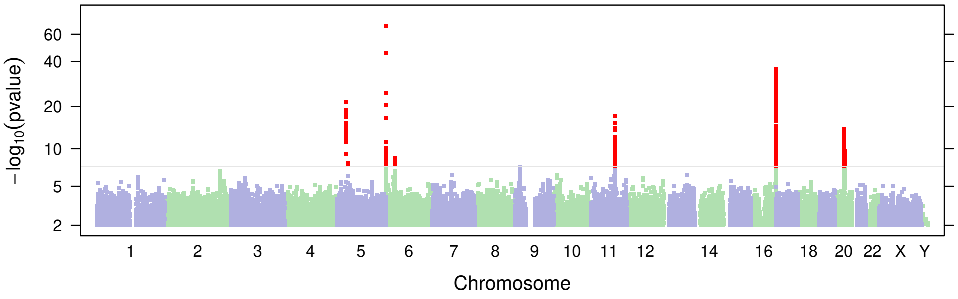

c) Melanoma

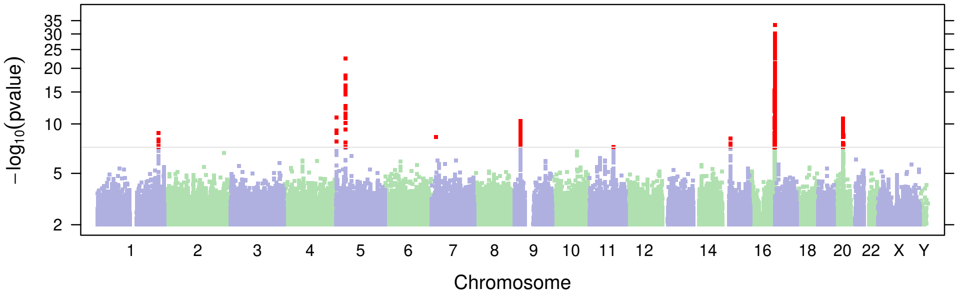

Supplementary Figure 6: Risk score performances in training and validation sets

a) and b) ROC (+ AUC) and precision−recall curves for the Disease Risk Score (DRS) in the training and validation sets. c), d) and e) are AUCs in the validation set for BCC, SCC, and melanoma, respectively. AUC for DRS, age, PRS, and Exposure risk score are shown. f), g) and h) are AUCs in the validation set across the participants age.

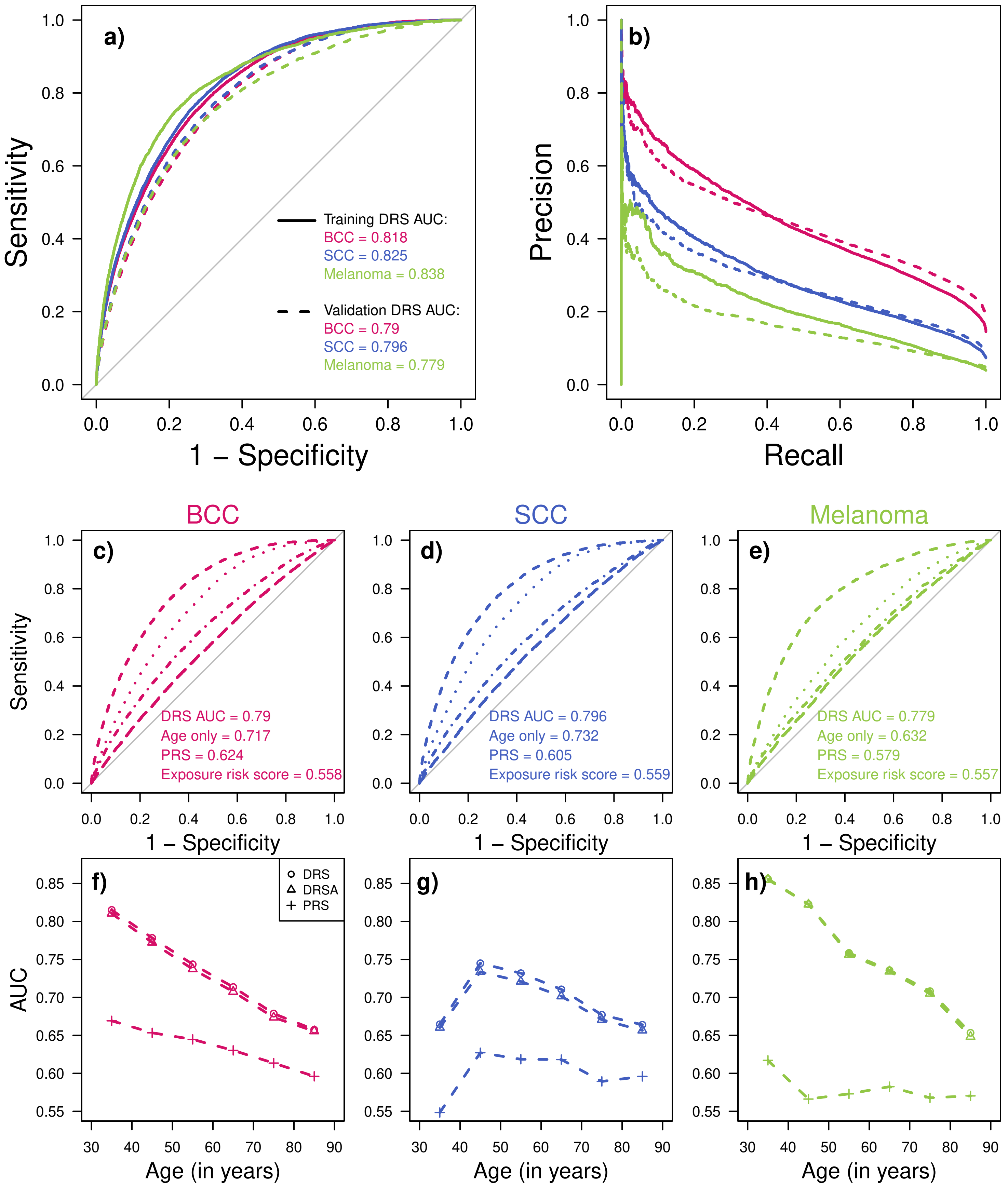

Supplementary Figure 7: Prevalence of skin cancer cases across the Demographic, Exposure, Susceptibility, and Mole risk score distributions in the validation set.

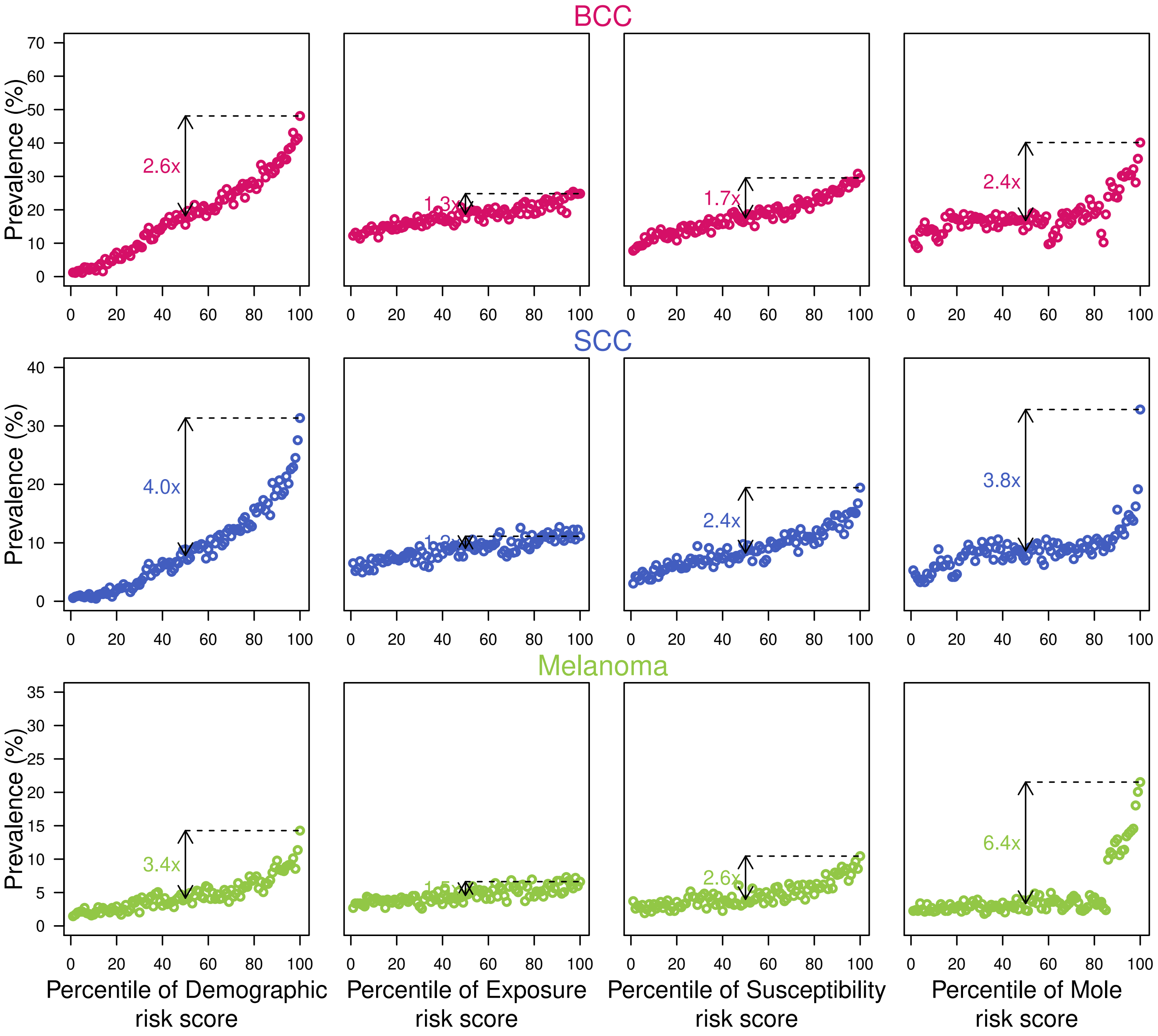

Supplementary Figure 8: DRS, DRSA and PRS distributions across the participant age at baseline in the validation set (103,008 participants, including 14,898 BCC, 7,479 SCC, and 3,998 melanoma cases).

Mean (dots) and SD (bars) of risk scores by age. The black area is the 95% confidence interval from linear models, with age modeled as polynomial.

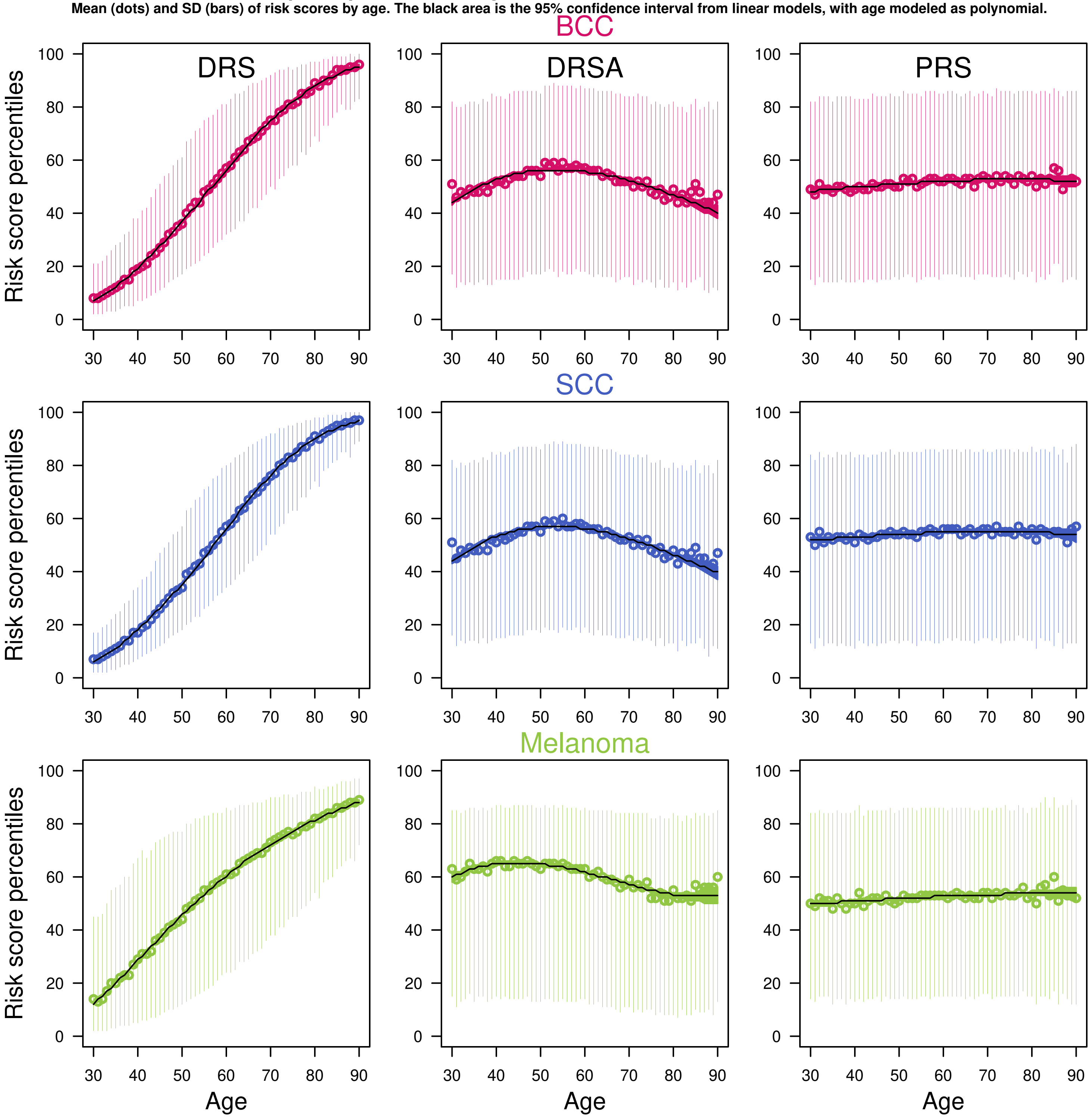

Supplementary Figure 9: Principal component analysis on PRS, Exposure, Susceptibility, Mole, Family, and Miscellaneous risk scores.

Right panel: all participants; Middle panel: skin cancer cases; Left panels: variance explained and legends of right and middle panels.

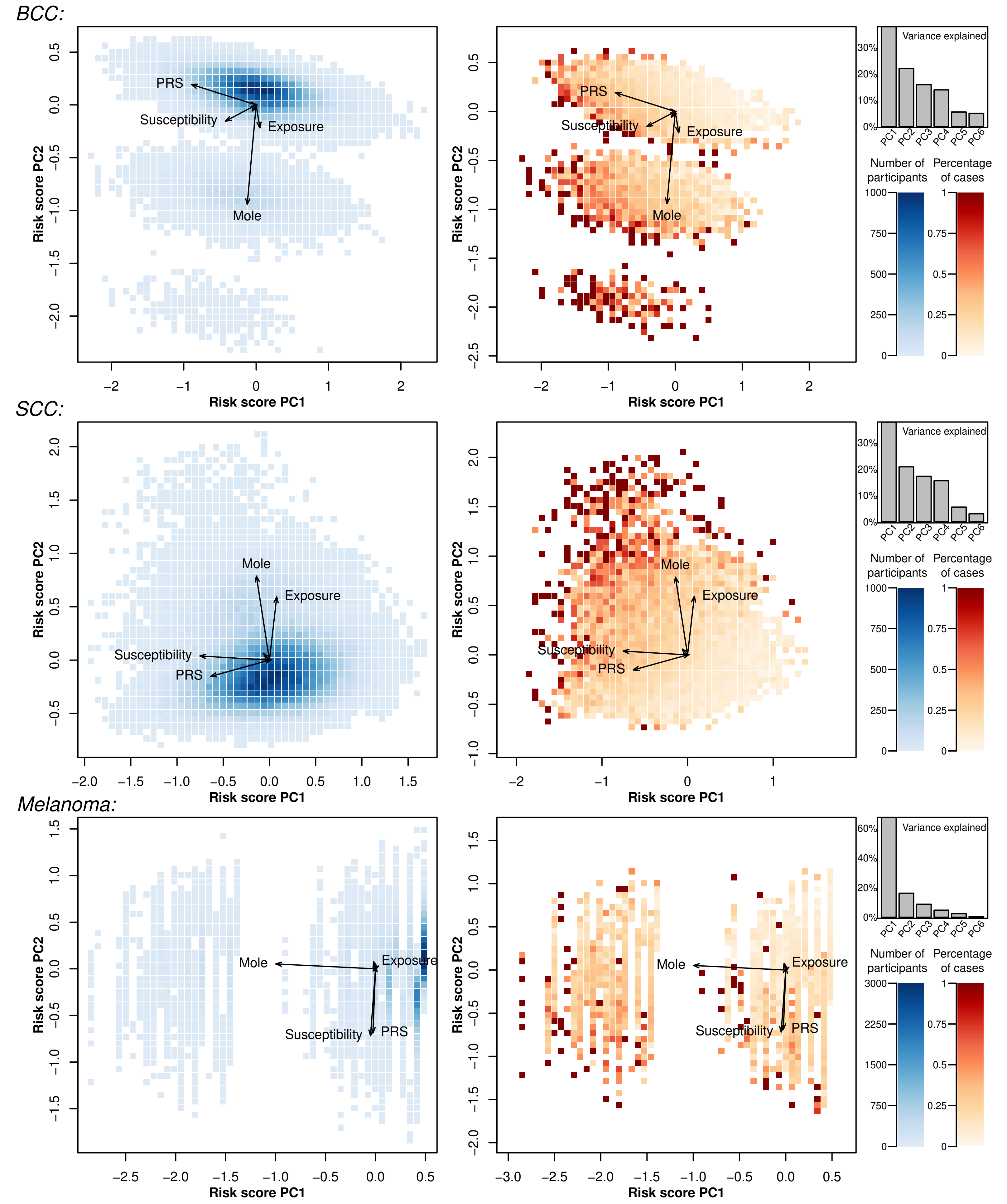

Supplementary Figure 10: Mean age (and 95% CI) of skin cancer diagnosis across the Demographic, Exposure, Susceptibility, and Mole risk score distributions in the validation set (103,008 participants, including 14,898 BCC, 7,479 SCC, and 3,998 melanoma cases).

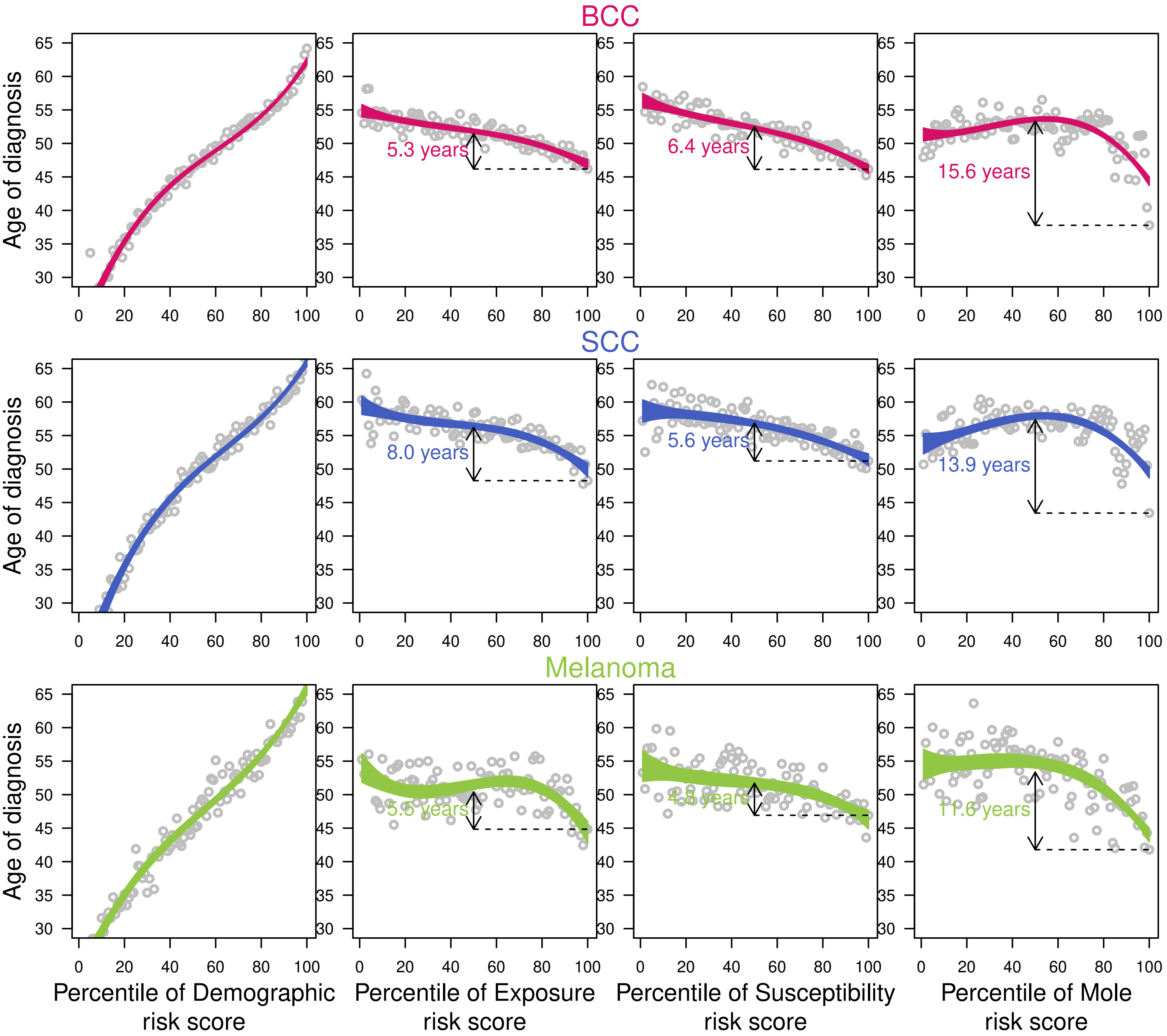

Supplementary Figure 11: Incident skin cancer cases in the validation set, from 2018 and 2019 followup survey.

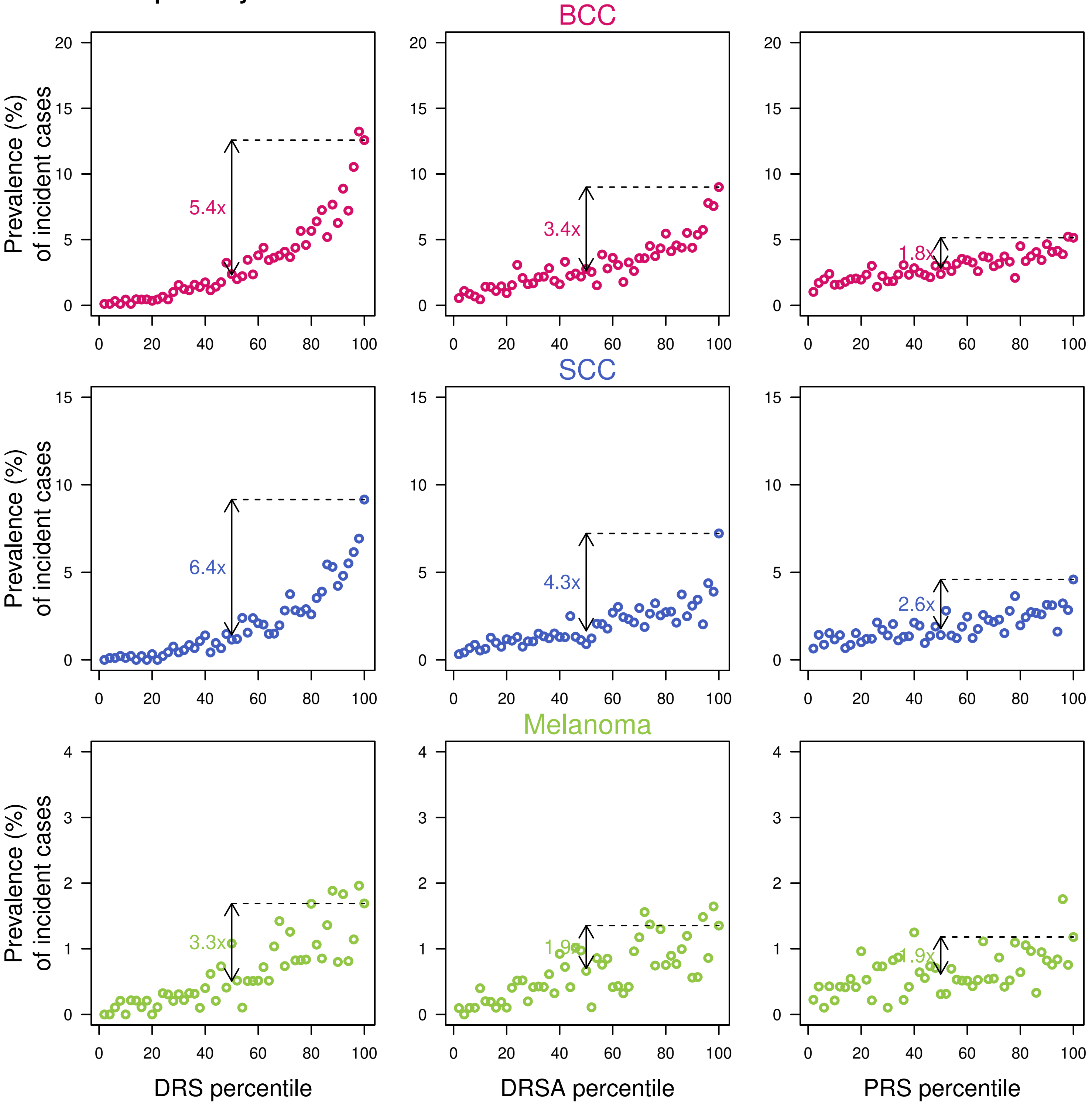

Supplementary Figure 12: Cancer free in the validation set, from 2018 and 2019 followup survey.

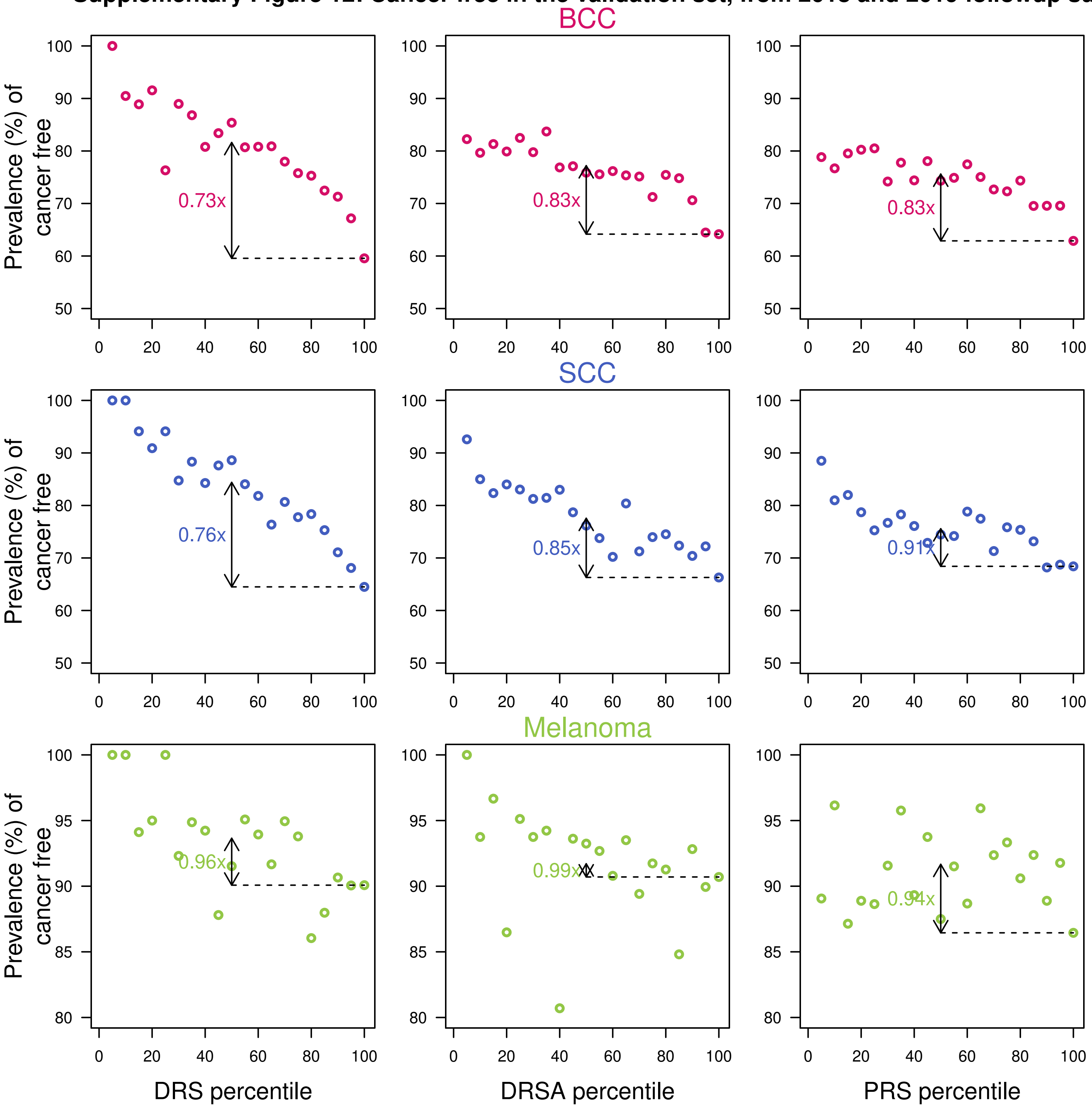

Supplementary Figure 13: Deviance explained in prediction models with pairwise interaction.

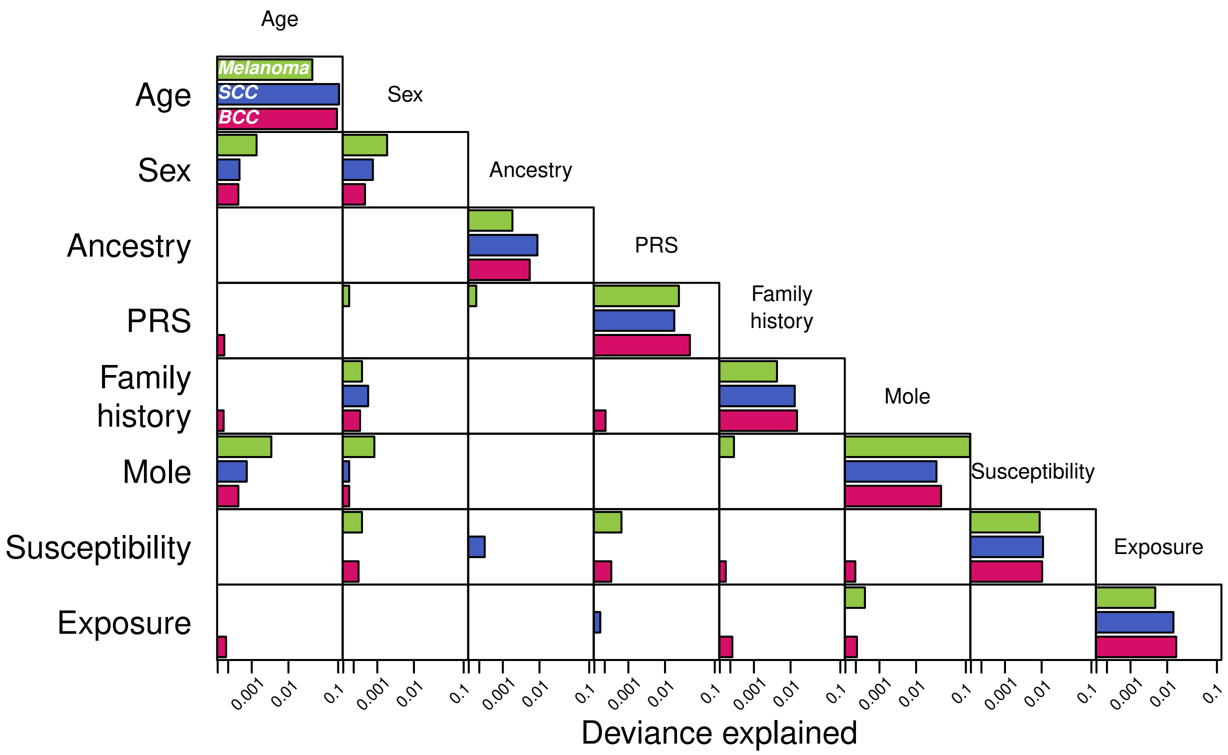

Supplementary Figure 14: Comparison between a published melanoma PRS (Gu et al. 2018) and the melanoma PRS from this study

Gu et al. (2018) developed a melanoma PRS using 12,874 melanoma cases and 23,203 controls from the Melanoma Meta−Analysis Consortium as a training set, and 3102 cases and 2301 controls from the MelaNostrum consortium for validation. The Melanoma Meta−Analysis Consortium combined multiple cohorts from Europe, Australia and the United States. Cases were identified through clinicians, pathology or cancer registers, and cases were generally diagnosed with pathology or histopathology confirmed invasive, cutaneous melanoma. The MelaNostrum Consortium included cases with histologically confirmed primary cutaneous melanoma from Italy, Spain, Greece and Cyprus. The PRS included 204 SNPs and achieved an AUC = 0.644 (95% CI = 0.630−0.658). All 204 variants were present in 23andMe database and passed our quality control (QC) requirements. a) and b) are a comparison of the estimated effects (and 95% CI) of the 204 PRS variants in 23andMe training set and in Gu et al. (2018)'s publication. The two figures present the variants with a P−value<1e−3 or >1e−3, respectively, in 23andMe training set. c) and d) are the performance of Gu et al.'s PRS in 23andMe validation set. The dashed line used directly Gu et al.'s PRS weights from the publication. The solid line used effect estimates from the 23andMe training set. e) Comparison between melanoma PRS from this study (grouped by percentile bins) and Gu et al. (2018) melanoma PRS (mean and SD of percentiles) in the validation set.

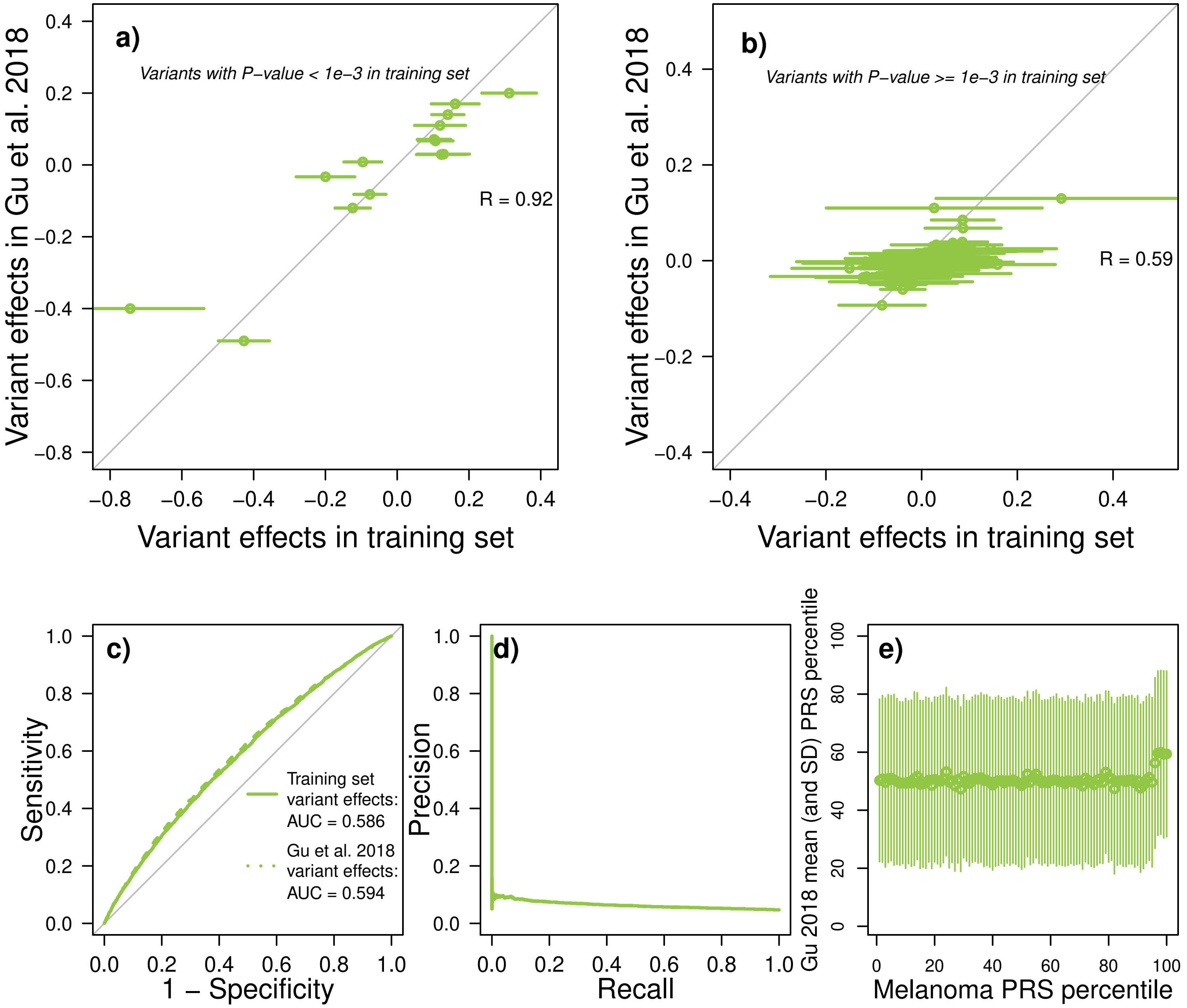

Supplementary Figure 15: Prevalence of skin cancer per 5% DRS bins and AUC in the validation set, split by European ancestries.

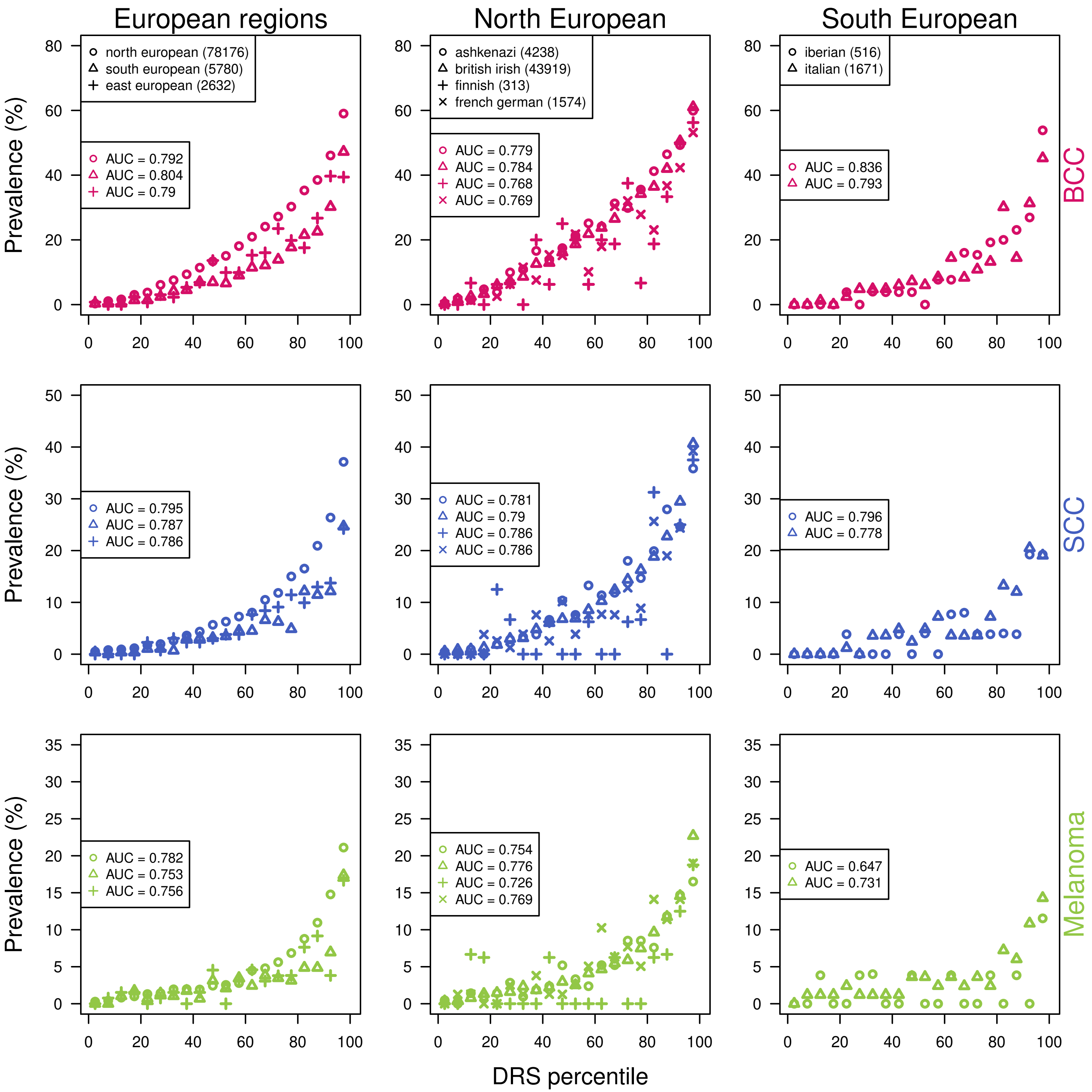

Supplementary Figure 16: Correlation matrix, in the training set, for the 32 factors included in the final models.

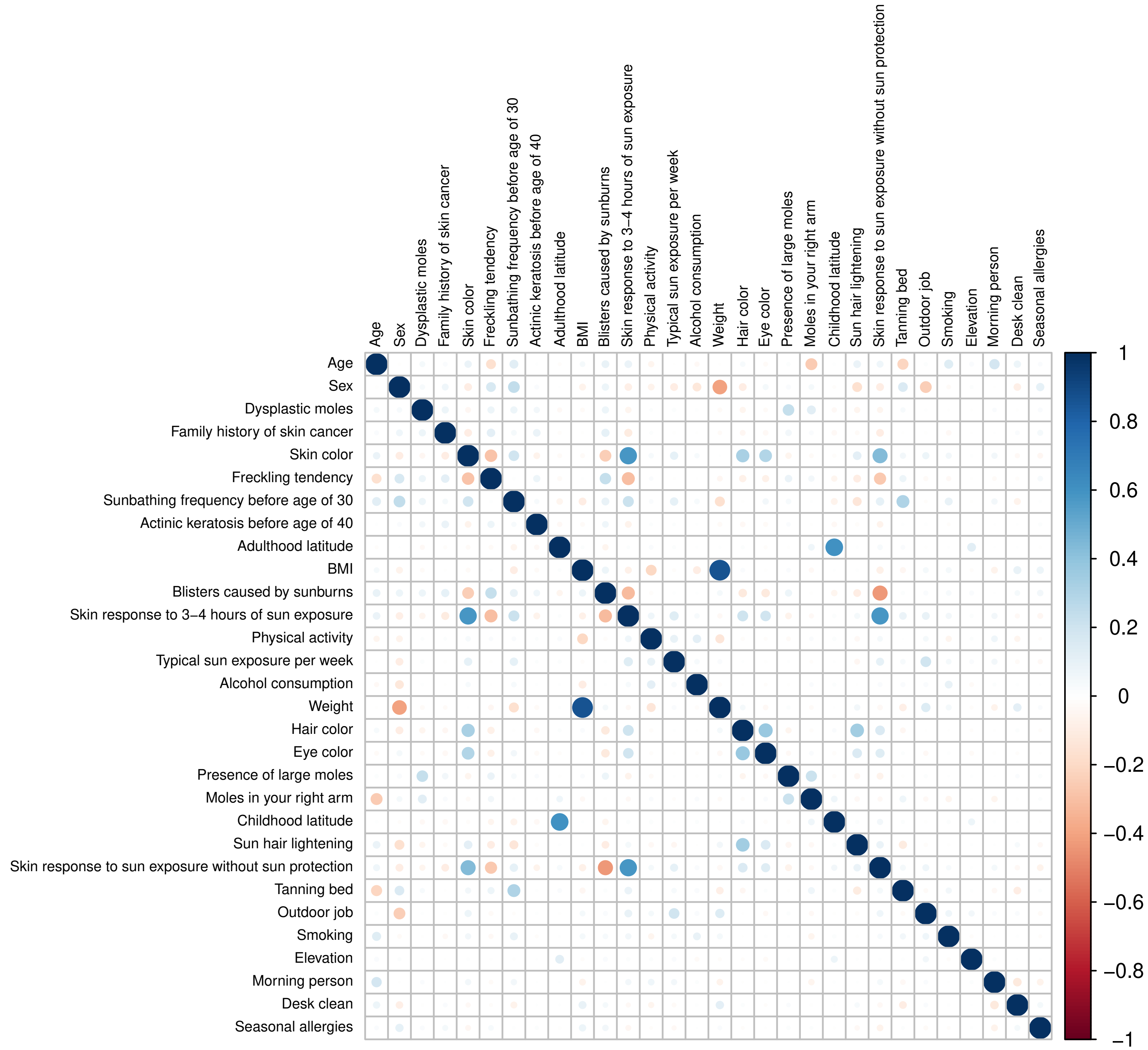

Supplementary Figure 17: Proportion of DRSA percentile bins in each age class.

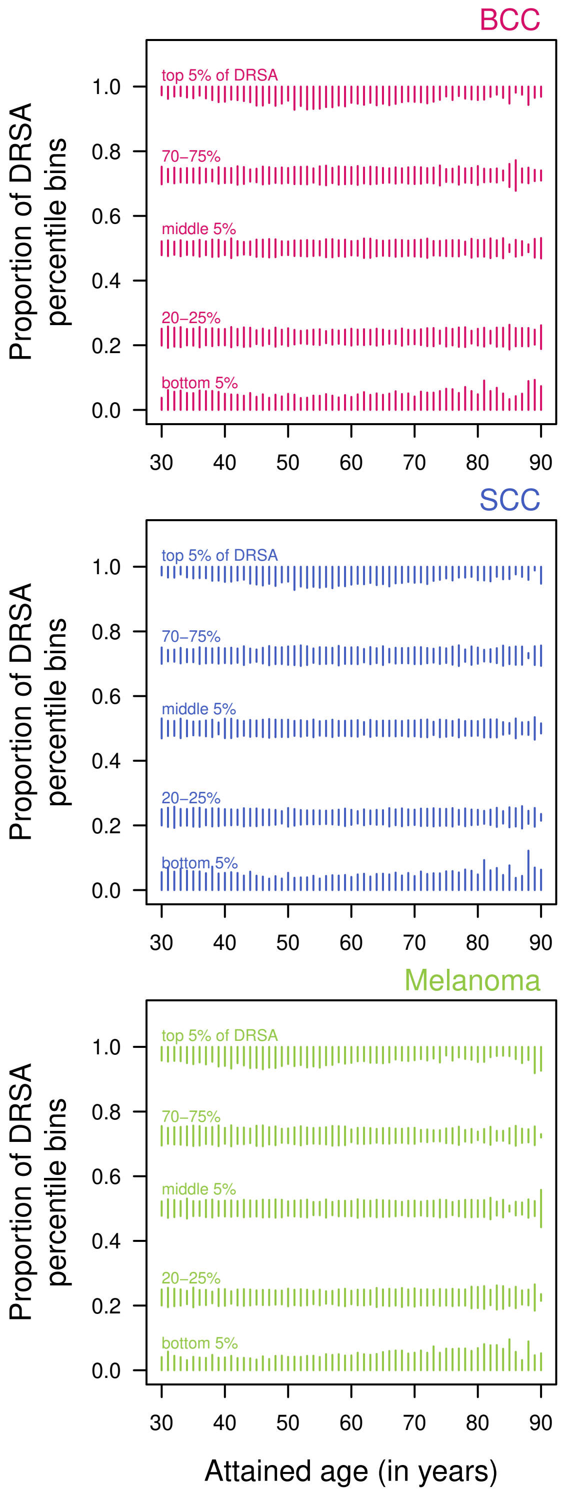

Supplementary Figure 18: Expected and observed incidence rate in the 2-year follow-up for the bottom, middle, and top 5% DRSA.

The expected incidence rates were computed from the prevalences from the validation set (see Methods). The observed incidence rates were calculated using the 2-year follow-up survey for participants with baseline bottom, middle, and top 5% DRSA (circle, triangle, and cross, respectively).

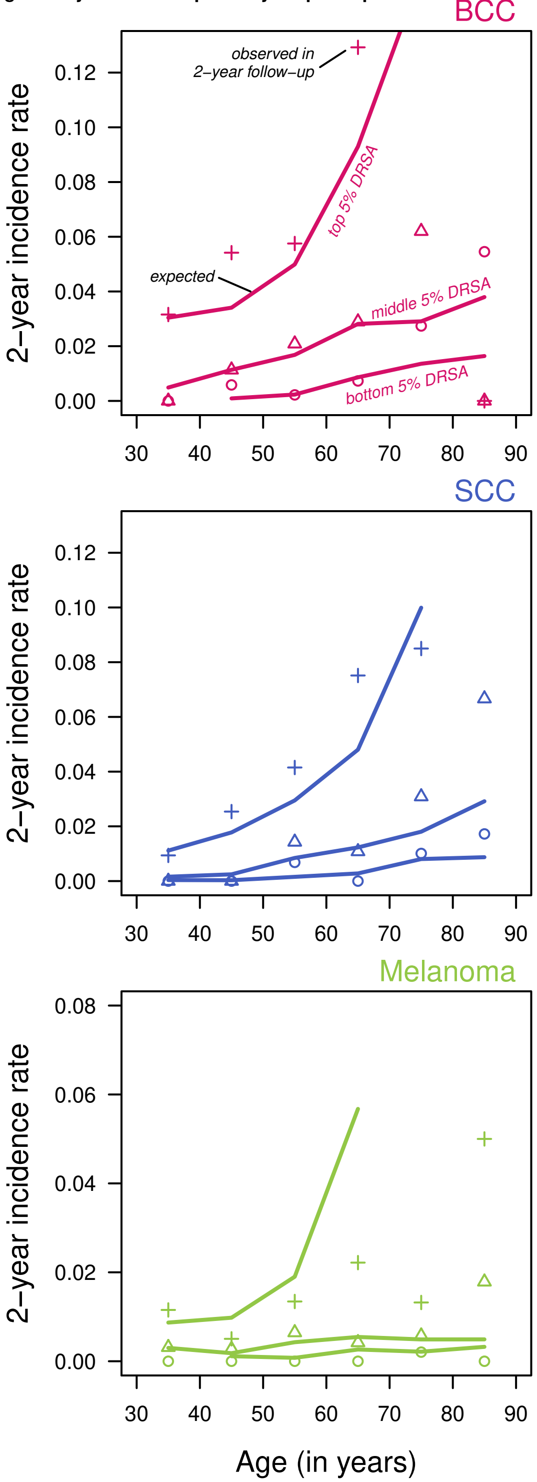

Supplementary Figure 19: Mean risk score contribution to DRSA scores across ages, for the bottom, middle, and top 5% DRSA.

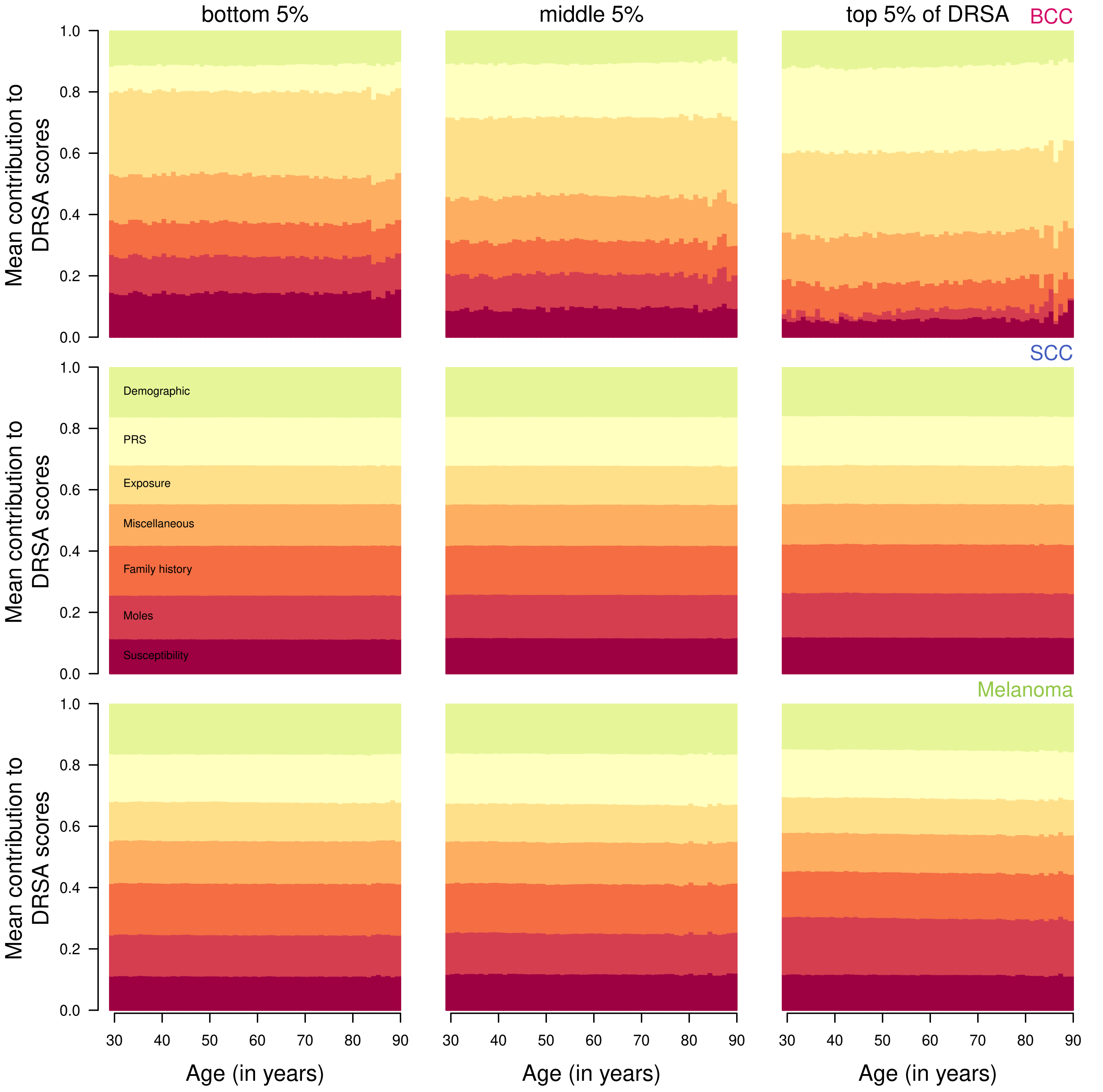

Supplement: Supplementary file 1 — Supplementary Information [file 41467_2020_20246_MOESM1_ESM.pdf]
