# Supplementary material for: Disease risk scores for skin cancers
Source: Nat Commun. 2021 Jan 8;12:160. doi: 10.1038/s41467-020-20246-5 (PMC7794415; doi:10.1038/s41467-020-20246-5)
Supplement: Supplementary file 3 — Reporting Summary [file 41467_2020_20246_MOESM3_ESM.pdf]

nature research

Corresponding author(s): Pierre Fontanillas

Last updated by author(s): Oct 1, 2020

Reporting Summary

Nature Research wishes to improve the reproducibility of the work that we publish. This form provides structure for consistency and transparency in reporting. For further information on Nature Research policies, see our Editorial Policies and the Editorial Policy Checklist.

Statistics

For all statistical analyses, confirm that the following items are present in the figure legend, table legend, main text, or Methods section.

n/a	Confirmed	
☐	☒	The exact sample size (*n*) for each experimental group/condition, given as a discrete number and unit of measurement
☐	☒	A statement on whether measurements were taken from distinct samples or whether the same sample was measured repeatedly
☐	☒	The statistical test(s) used AND whether they are one- or two-sided<br>*Only common tests should be described solely by name; describe more complex techniques in the Methods section.*
☐	☒	A description of all covariates tested
☐	☒	A description of any assumptions or corrections, such as tests of normality and adjustment for multiple comparisons
☐	☒	A full description of the statistical parameters including central tendency (e.g. means) or other basic estimates (e.g. regression coefficient) AND variation (e.g. standard deviation) or associated estimates of uncertainty (e.g. confidence intervals)
☐	☒	For null hypothesis testing, the test statistic (e.g. *F*, *t*, *r*) with confidence intervals, effect sizes, degrees of freedom and *P* value noted<br>*Give P values as exact values whenever suitable.*
☒	☐	For Bayesian analysis, information on the choice of priors and Markov chain Monte Carlo settings
☒	☐	For hierarchical and complex designs, identification of the appropriate level for tests and full reporting of outcomes
☐	☒	Estimates of effect sizes (e.g. Cohen's *d*, Pearson's *r*), indicating how they were calculated

Our web collection on statistics for biologists contains articles on many of the points above.

Software and code

Policy information about availability of computer code

Data collection	Phenotype data were collected by online questions and surveys on the 23andMe Research platform (https://customercare.23andme.com/hc/en-us/articles/212881977-23andMe-Research-Surveys-and-Questions) from consented 23andMe Research Participants (https://customercare.23andme.com/hc/en-us/articles/212195708-Research-Participation-and-Consent).<br>Genetic data were the de-identified individual-level genetic Information of the consented 23andMe Research Participants (https://www.23andme.com/howitworks/).
Data analysis	Genetic and GWAS analyses where performed on 23andMe Research platform. It has been described in numerous peer-reviewed publications (https://research.23andme.com/publications/).<br>Additional statistical analyses, figures, and tables were generated on R v3.3.

For manuscripts utilizing custom algorithms or software that are central to the research but not yet described in published literature, software must be made available to editors and reviewers. We strongly encourage code deposition in a community repository (e.g. GitHub). See the Nature Research guidelines for submitting code & software for further information.

Data

Policy information about availability of data

All manuscripts must include a data availability statement. This statement should provide the following information, where applicable:

- Accession codes, unique identifiers, or web links for publicly available datasets
- A list of figures that have associated raw data
- A description of any restrictions on data availability

23andMe provides access to full summary statistics from published GWAS analyses through a Data Transfer Agreement that protects the privacy of our participants'

data (https://research.23andme.com/dataset-access/).
The summary statistics for the phenotype data were included in the main manuscript and the supplementary material.

Field-specific reporting

Please select the one below that is the best fit for your research. If you are not sure, read the appropriate sections before making your selection.

☒ Life sciences ☐ Behavioural & social sciences ☐ Ecological, evolutionary & environmental sciences

For a reference copy of the document with all sections, see nature.com/documents/nr-reporting-summary-flat.pdf

Life sciences study design

All studies must disclose on these points even when the disclosure is negative.

Sample size

The sample size was determined based on GWAS and PRS statistical powers.

Data exclusions

Genetic data was processed through 23andMe GWAS pipeline; Genotyped and imputed variants were excluded based on Quality Control statistics.
Only the phenotype data of 23andMe participants who completed the full surveys and questions were included in the analyses. No other exclusion criteria were used.

Replication

We included in the main manuscript and supplementary material comparisons to published and independent datasets.

Randomizatio

The manuscript included Demographic statistic comparison between the training and validation sets.

Blinding

Group allocation for the training and validation sets was determined by the date of online survey completion. After consenting to research, 23andMe Research Participants can complete the surveys at anytime.

Reporting for specific materials, systems and methods

We require information from authors about some types of materials, experimental systems and methods used in many studies. Here, indicate whether each material, system or method listed is relevant to your study. If you are not sure if a list item applies to your research, read the appropriate section before selecting a response.

Materials & experimental systems

n/a	Involved in the study	
☒	☐	Antibodies
☒	☐	Eukaryotic cell lines
☒	☐	Palaeontology and archaeology
☒	☐	Animals and other organisms
☐	☒	Human research participants
☒	☐	Clinical data
☒	☐	Dual use research of concern

Methods

n/a	Involved in the study	
☒	☐	ChIP-seq
☒	☐	Flow cytometry
☒	☐	MRI-based neuroimaging

Human research participants

Policy information about studies involving human research participants

Population characteristics

The cohort used in this study is restricted to 23andMe Research Participants from European ancestry. Ancestry for 23andMe Research Participants was determined via genetics.
The demographic characteristics of this cohort was described in the main manuscript and the supplementary material.

Recruitment

The 23andMe Research cohort is derived from the 23andMe Customers. Every 23andMe Research Participants had the opportunity to complete the online surveys (non-targeted surveys).

Ethics oversight

23andMe Research Participants provided informed consent and participated in the research online, under a protocol approved by the external AAHRPP-accredited IRB, Ethical & Independent Review Services (www.eandireview.com).

Note that full information on the approval of the study protocol must also be provided in the manuscript.
